# Composition and Functional State of T and NK Cells in the Extramedullary Myeloma Tumor Microenvironment

**DOI:** 10.1158/2643-3230.BCD-25-0170

**Published:** 2025-11-14

**Authors:** Anjana Anilkumar Sithara, Veronika Kapustova, David Zihala, Ondrej Venglar, Daniel Bilek, Moutaz Helal, Mara John, Eva Radova, Lucie Broskevicova, Jan Vrana, Gabriela Havlova, Ludmila Muronova, Tereza Popkova, Jana Mihalyova, Hana Plonkova, Serafim Nenarokov, Kamlesh Bisht, Hongfang Wang, Helgi Van de Velde, Sandra Charvatova, Ivo Demel, Michal Kascak, Milan Navratil, Martin Havel, Juli Bago, Michal Simicek, Angela Riedel, Leo Rasche, Tereza Sevcikova, Ola Landgren, Roman Hajek, Tomas Jelinek

**Affiliations:** 1 Department of Hematooncology, University Hospital Ostrava, Ostrava, Czech Republic.; 2 Department of Hematooncology, Faculty of Medicine, University of Ostrava, Ostrava, Czech Republic.; 3 Department of Biology and Ecology, Faculty of Science, University of Ostrava, Ostrava, Czech Republic.; 4 Mildred Scheel Early Career Center, University Hospital Würzburg, Würzburg, Germany.; 5 Department of Nuclear Medicine, University Hospital Ostrava, Ostrava, Czech Republic.; 6 Institute of Parasitology, Biology Centre, Czech Academy of Sciences, České Budějovice, Czech Republic.; 7 Research and Development, Sanofi, Cambridge, Massachusetts.; 8 Department of Internal Medicine II, University Hospital Würzburg, Würzburg, Germany.; 9 Sylvester Myeloma Institute, Sylvester Comprehensive Cancer Center, University of Miami, Miami, Florida.

## Abstract

Extramedullary multiple myeloma (EMM) is a high-risk feature of multiple myeloma associated with increased resistance to treatments, including modern immunotherapies, and shorter survival. The composition and functional state of immune cells within the EMM tumor microenvironment (TME) remain poorly understood. Using single-cell RNA sequencing, flow cytometry, and spatial transcriptomics, we revealed significant differences in the EMM TME compared with multiple myeloma bone marrow (BM). T and NK cells were verified as the most abundant immune subsets in the EMM TME. Compared with the BM counterparts, we found these tumors to have a significantly reduced effector–to–tumor cell ratio, a significantly lower number of CD4^+^ T cells, and an increased proportion of regulatory CD16^−^ NK cells. We observed a high proportion of exhausted, tumor-reactive CD8^+^ T cells in roughly half of EMM tumors. Furthermore, we identified elevated expression of immune checkpoints, such as PD-1 on CD8^+^ T cells and *KLRC1* (*NKG2A*) on CD16^−^ NK cells.

**Significance:**

This study characterizes the TME in EMM lesions and paired BM from patients with multiple myeloma, revealing a high proportion of less cytotoxic CD16^−^ NK cells in EMM tumors and suggesting that direct cell–cell interactions may underlie the CD8^+^ T-cell exhaustion observed in a subset of these tumors.

## Teaser

Analysis of extramedullary myeloma (EMM) lesions and paired bone marrow samples reveals low immune infiltration in the EMM microenvironment, with enrichment of CD16^−^ NK cells and of CD8^+^ T cell exhaustion phenotypes.

## Introduction

Extramedullary disease (EMD) represents a high-risk clinical feature for patients with multiple myeloma, caused by aggressive multiple myeloma clones egressing from the bone marrow (BM) and effectively colonizing new niches in the body (1). Typically, EMD is more commonly seen in patients with relapsed/refractory multiple myeloma (RRMM), for which the incidence rate of developing secondary EMD (which develops during multiple myeloma treatment) is as high as 43% (compared with approximately 4.5% at diagnosis; primary EMD; refs. 2, 3). Importantly, extramedullary multiple myeloma (EMM) cells exhibit higher resistance to both traditional agents (4) and modern immunotherapies such as naked monoclonal antibodies (mAb; ref. 5), bispecific antibodies (bsAb; refs. 6–11), and chimeric antigen receptor (CAR) T cells (12–14). It is important to distinguish extramedullary lesions from paramedullary diseases (PMD), which are adjacent to the bone. Many studies report PMD as EMM, but only EMMs characterized by soft-tissue plasmacytomas that are truly independent of the bone are associated with a significantly worse prognosis (4, 15).

Prior studies suggest that the aggressive nature of EMM may be attributed to tumor-intrinsic factors such as increased proliferation (16–18) and a higher mutational burden compared with multiple myeloma cells in BM, enhancing the evolutionary plasticity of EMM cells and leading to the emergence of treatment-resistant subclones (18–20). Another contributing factor could be a specific genetic makeup associated with EMM, characterized by the co-occurrence of MAPK pathway mutations (e.g., *KRAS*, *NRAS*, *BRAF*; refs. 21, 22) together with 1q gain/amplification (23, 24), which has been observed in the majority of EMM tumors and is often present already at multiple myeloma diagnosis (18, 20, 25). Downstream, these changes likely contribute to the observed decreased expression of relevant therapeutic targets (e.g., CD38, GPRC5D), as well as proteins of the MHC I complex, which are essential for the optimal recognition of malignant cells by immune effector cells (5, 18, 26).

Current multiple myeloma treatment strategies increasingly leverage immunotherapy to activate the patient’s immune system, particularly T and NK effector cells (27, 28), to fight the tumor. It is well established that T cells are crucial for bsAb efficacy, as they mediate targeted tumor cell killing, whereas NK cells play a key role in the activity of anti-CD38 mAbs through antibody-dependent cellular cytotoxicity (ADCC; ref. 29). Notably, T and NK cells consist of diverse subsets with distinct functional states, potentially influencing therapeutic outcomes. Specifically, among NK cell subtypes, the CD16^+^CD56^−^ subset exhibits potent cytotoxic activity, whereas CD16^−^CD56^+^ cells, which typically represent around 10% of NK cells in the blood and BM of patients with multiple myeloma (30), primarily produce cytokines and possess lower cytotoxic capacity due to the absence of CD16 (FcγRIII), a key receptor for ADCC (31, 32), highlighting their distinct roles in tumor surveillance. Although previous studies have reported a relatively low abundance of tumor-infiltrating lymphocytes (18) and some degree of CD8^+^ T-cell exhaustion within the EMM microenvironment (12, 19), a direct comparison of the composition and functional status of immune effector cells between EMM and BM multiple myeloma remains lacking, to the best of our knowledge.

**Figure 1.**
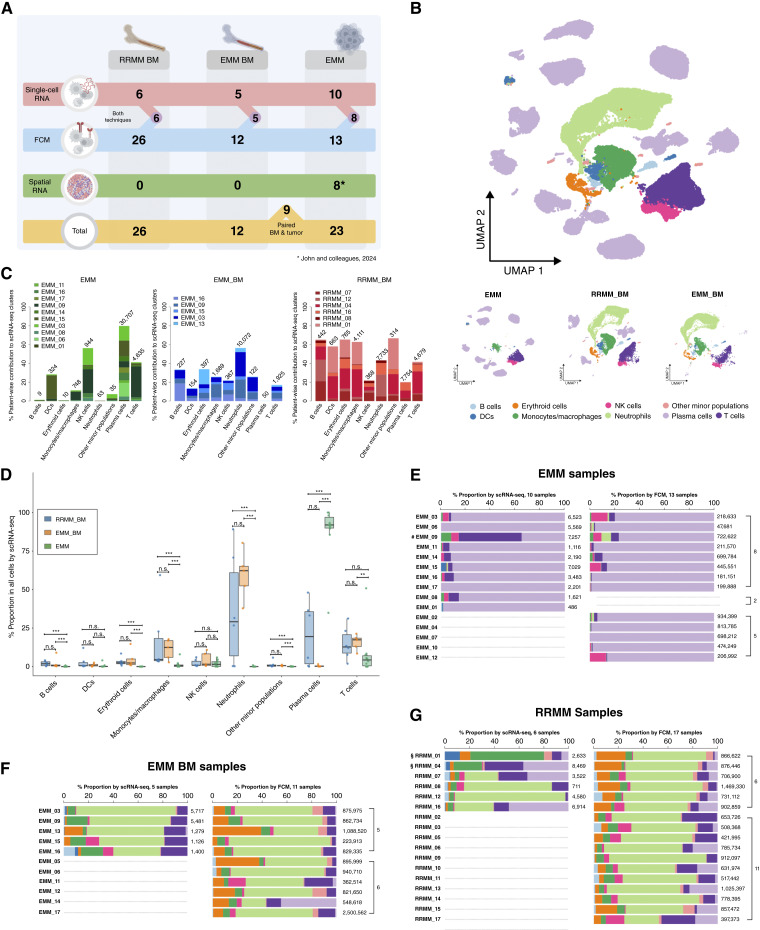
Study design and the overall composition of EMM tumors and the multiple myeloma BM microenvironment. **A,** Study design depicting the total number of samples and the techniques used. Vertical connections between lines highlight how many patients were analyzed by both scRNA-seq and FCM. **B,** UMAP representation of all cells in the scRNA-seq cohort, displaying 79,307 cells in total, annotated into nine different cell types marked by different colors and separate UMAP representations for each sample type displaying cells in EMM (*N* = 10), EMM_BM (*N* = 5), and RRMM_BM (*N* = 6). **C,** Stacked bar plot showing patient-wise contribution to scRNA-seq clusters. The number above each bar indicates the total number of cells in the corresponding cluster. The proportions sum to 100% across EMM, EMM_BM, and RRMM samples. **D,** Box plot showing the proportions of major cell subtypes identified by scRNA-seq, grouped by sample type (RRMM_BM, EMM_BM, and EMM), with colors indicating the respective groups. The stacked bar plot indicates the proportion of different cell types in each sample identified by scRNA-seq (left) and FC (right) for (**E**) EMM, (**F**) EMM_BM, and (**G**) RRMM cohorts. The number above each bar indicates the total number of cells in the corresponding sample, with cell annotations color-coded in (**B**). #, a sample that was artificially enriched for infiltrating immune cells as described in the “Methods” section “scRNA-Seq”; §, the absence of neutrophils in the corresponding FCM data is likely due to a delay in sample processing for scRNA-seq, as described in detail in Supplementary Fig. S11. Box plots display the median (center line), the 25th and 75th percentiles (box limits), and whiskers extending to the most extreme data points within 1.5× the IQR. Statistical comparisons were performed using the Wilcoxon rank-sum test with Benjamini–Hochberg correction for multiple testing. n.s., not significant; **, *P* < 0.05; ***, *P* < 0.01. DC, dendritic cell.

In this study, we conducted a comprehensive single-cell analysis of patients with EMM tumors (*N* = 23; Fig. 1A), including unique BM samples from patients with EMM (EMM_BM; *N* = 12, of which nine BM samples were paired with EMM samples), as well as RRMM BM samples from independent patients without evidence of EMM (*N* = 26, RRMM_BM). A combination of three single-cell techniques was used, including single-cell RNA sequencing (scRNA-seq), flow cytometry (FCM), and spatial transcriptomics (ST). This approach uncovered crucial differences in the tumor microenvironment (TME) of EMM tumors and BM, which may help guide the development of more effective treatment strategies for EMM in the future.

## Results

### Patients’ Characteristics

EMM and RRMM cohorts were well balanced in terms of sex, international staging system (ISS) stage at diagnosis, previous lines of therapy, and other clinical features (Supplementary Tables S1 and S2) and treatment (Supplementary Tables S3 and S4). However, patients with EMM were markedly younger at the time of sampling (median 59 vs. median 70 years). Furthermore, in patients with EMM, we observed significantly lower levels of BM plasma cells (BMPC) at the time of relapse, as measured by FCM (according to our clinical standards described in Venglar and colleagues; ref. 33) and by cytologic analysis (Supplementary Tables S1 and S2). Conversely, lactate dehydrogenase (LDH) levels at relapse were significantly higher in the EMM cohort (4.57 vs. 3 μkat/L, *P* < 0.01). Importantly, the clinical characteristics of the scRNA-seq and FCM cohorts were balanced for both EMM (10 scRNA-seq and 16 FCM samples; Supplementary Table S5) and patients with RRMM (6 scRNA-seq and 26 FCM samples; Supplementary Table S6). To validate the scRNA-seq and FCM data, spatial data analysis of an independent cohort of eight patients with EMM was included, which was described previously (19).

### Overall Composition of EMM Tumors and Multiple Myeloma BM Microenvironment

scRNA-seq of 122,932 cells from EMM (*N* = 10), EMM_BM (*N* = 5; 4 paired samples), and RRMM_BM (*N* = 6) yielded a total number of 79,307 high-quality cells following initial preprocessing and stringent quality control steps (detailed in the “Methods” section “scRNA-Seq Data Analysis: Preprocessing”). This includes sample EMM_09, which was subjected to an additional enrichment of immune cells as described in the “Methods” section “scRNA-Seq.” Substantiating our previous findings (18), EMM tumors were predominantly composed of malignant plasma cells (PC), T cells, and NK cells (Fig. 1B–E), represented by median proportions of 91.9%, 4.3%, and 1.5% of total nucleated cells, respectively (Fig. 1D). The infiltration by myeloid cells was minimal (median proportion of 0.9% of total nucleated cells) in the EMM cohort. Conversely, BM biopsies from patients with EMM showed minimal infiltration of malignant PCs, reflected by a median proportion of 0.2% of total nucleated cells. The dominant populations in EMM_BM samples were neutrophils, T cells, monocytes/macrophages, erythroid precursors, and NK cells (median proportions of 62.2%, 17.3%, 12.4%, 2.4%, and 1.5% of total nucleated cells, respectively; Fig. 1D and F). For comparison, samples from the RRMM cohort without evidence of EMM consisted mainly of neutrophils, T cells, monocytes/macrophages, and erythroid cells (median proportions of 29.1%, 12.7%, 4.6%, and 2.5% of total nucleated cells, respectively) and malignant PCs at a median proportion of 19.5% of total nucleated cells (Fig. 1D and G).

Consistent with the observations from scRNA-seq data, FCM analysis demonstrated malignant PCs, T cells, and NK cells (median proportions of 95.9%, 0.9%, and 0.9% of total nucleated cells, respectively; Fig. 1E; Supplementary Table S7) as major constituents of EMM tumor biopsies (*N* = 13), whereas BM samples from patients with EMM (*N* = 11) had very low to no malignant PC infiltration (median proportion of 0.5% of total nucleated cells; Fig. 1F; Supplementary Table S7). EMM_BM samples were composed mainly of neutrophils, with a median of 62.3%, followed by erythroid cells, T cells, monocytes/macrophages, and NK cells (median proportions of 9%, 8.5%, 5.7%, and 2.8% of total nucleated cells, respectively; Fig. 1F; Supplementary Table S7).

In summary, FCM and scRNA-seq methods yielded comparable results regarding the proportions of each cell type (Fig. 1E–G; Supplementary Figs. S1A, S1B, and S2A–S2D), consistently revealing that EMM tumors are primarily composed of malignant PCs. The effector/tumor (E:T) ratio was defined by the ratio of effector cells (NK and T cells) to malignant PCs. The E:T ratio was substantially lower in EMM compared with BM samples (FCM median 0.03, 17.5, and 2.1 for EMM, EMM_BM, and RRMM, respectively; *P* < 0.01; Supplementary Fig. S3A), and malignant PC infiltration in EMM_BM was minimal in our cohort (median proportion of 0.2% of total nucleated cells by scRNA-seq and 0.5% by FCM; Fig. 1F). As expected, scRNA-seq data also revealed a higher proportion of malignant PCs in the S–G2–M phase in EMM compared with RRMM_BM samples (Supplementary Fig. S4A), further supported by the enriched pathways associated with proliferation, including E2F_targets and G_2_–M checkpoints from the Hallmark gene sets (Supplementary Fig. S4B).

### scRNA-seq Identifies a Diverse Range of T-cell and NK-cell Subsets

**Figure 2.**
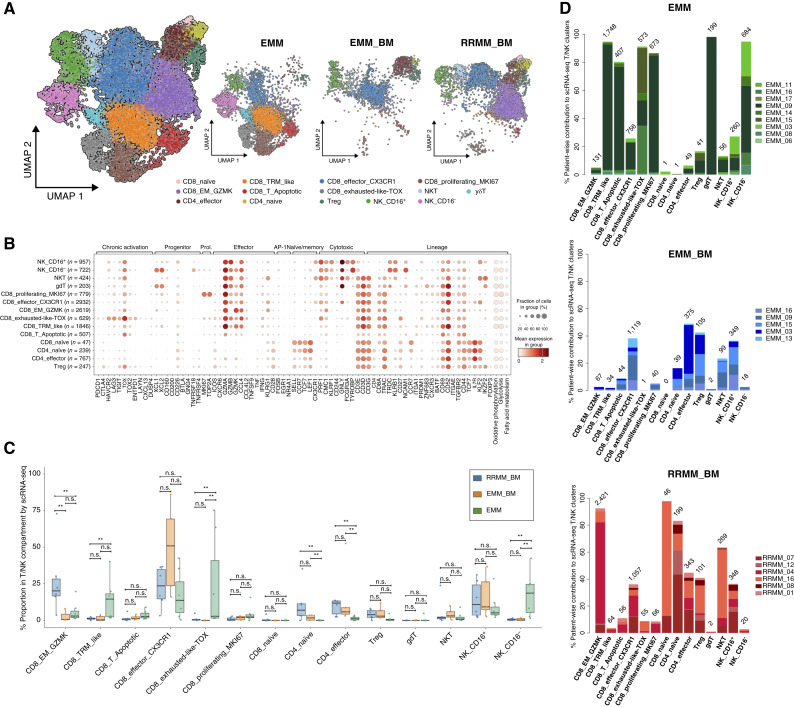
Diverse subsets of T and NK cells identified by scRNA-seq. **A,** UMAP of T/NK subclusters, displaying a total of 12,918 cells, grouped into 14 subclusters marked by color codes (left). UMAP of T/NK subclusters separated for each sample type, displaying cells in EMM (*N* = 7), EMM_BM (*N* = 5), and RRMM_BM (*N* = 6), with the colors representing the same cell type as in the global UMAP of the T/NK compartment (right). **B,** Dot plot highlighting the expression of canonical markers and gene signatures defining the subclusters in the T/NK compartment. The marker list was curated using Friedrich and colleagues (35), and the genes for defining the signatures are adopted from Chu and colleagues (59). **C,** Box plot representation of the composition of cell subtypes in the T/NK compartment, grouped by sample type and marked with different colors. **D,** Stacked bar plots representing patient-wise contributions to individual T/NK subclusters. The proportions sum to 100% across EMM, EMM_BM, and RRMM samples. Box plots display the median (center line), the 25th and 75th percentiles (box limits), and whiskers extending to the most extreme data points within 1.5× the IQR. Statistical comparisons were performed using the Wilcoxon rank-sum test with Benjamini–Hochberg correction for multiple testing. n.s., not significant; **, *P* < 0.05; ***, *P* < 0.01; ****, *P* < 0.001.

Next, we aimed to further dissect the T/NK cell cluster from scRNA-seq data (*N* = 18). A semiautomated approach utilizing graph-based unsupervised Leiden clustering, CellTypist (34), and expression of canonical immune markers (see “Methods” sections “scRNA-Seq Data Analysis: Preprocessing” and “scRNA-Seq Cluster Annotation”) identified 14 distinct subclusters in the T/NK cell compartment (Fig. 2A and B): 12 clusters of T cells and two distinct clusters of NK cells categorized as CD16^+^ cells, which had reduced *NCAM1* (*CD56*) expression, and CD16^−^ cells with higher *NCAM1* (*CD56*) expression. Compared with CD8^+^ T and NK cells discussed below, we identified only a limited infiltration by CD4^+^ T cells, represented mostly by regulatory T cells (Treg), CD4 effector, and CD4 naïve T cells (Fig. 2C and D). Additionally, we tested the robustness of these observations by performing downsampling of T and NK cells to 122 cells to account for the variable number of cells contributed by patients and observed congruent results (Supplementary Fig. S5A).

### CD8^+^ T Cells from EMM Tumors Are Often More Exhausted Compared with Their BM Counterparts

Previously, it was shown that CD8^+^ T cells colocalizing with malignant PCs in EMM tumors express higher levels of exhaustion markers *HAVCR2* (TIM-3) and *PDCD1* (PD-1; ref. 19). However, it remains unclear how these cells compare with their BM multiple myeloma counterparts from the same patient. To address this question, we used paired BM samples collected at the time of EMM, enabling a direct comparison of the functional states of CD8^+^ T-cell subsets at both tumor locations.

**Figure 3.**
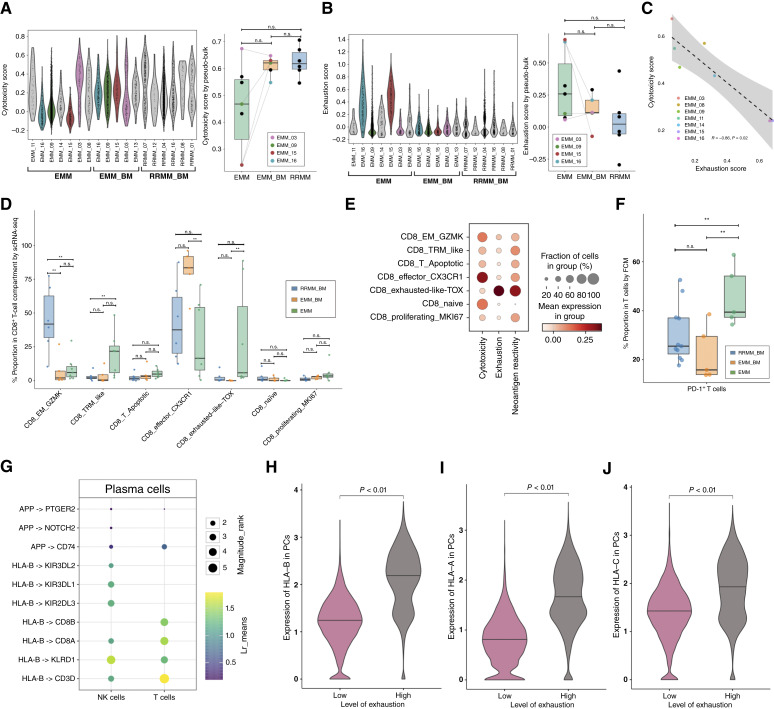
Levels of cytotoxicity, exhaustion, and neoantigen reactivity scores suggesting direct cell–cell interactions between EMM cells and CD8^+^ T cells. **A,** Violin plots indicating the T-cell cytotoxicity score in CD8^+^ T cells with marked divisions to classify samples by their sample types. The scores were computed using a curated gene list (35) and scRNA-seq data. Paired samples in the EMM and EMM_BM cohorts are highlighted in the same color (left). Box plot of cytotoxicity scores calculated using the same set of genes and estimated with pseudo-bulk and ssGSEA (right). **B,** Exhaustion score in CD8^+^ T cells with marked divisions to classify samples by their types (left). Box plot of exhaustion scores calculated with pseudo-bulk and ssGSEA, using scRNA-seq data (right). Figures are represented in the same format as in (**A**). **C,** Spearman correlation between exhaustion and cytotoxicity scores calculated from pseudo-bulk in scRNA-seq EMM samples. **D,** Box plot indicating the composition of cell subtypes in the CD8^+^ T-cell compartment. Each color here represents a sample type as mentioned in the legend. **E,** Dot plot showing average cytotoxicity and exhaustion scores and neoantigen reactivity in CD8^+^ T cells across the subclusters from all samples [EMM (*N* = 7), EMM_BM (*N* = 5), RRMM_BM (*N* = 6)], using scRNA-seq data. **F,** Box plot showing the proportion of cells with positive expression of the *PDCD1* (*PD-1*) gene relative to all the T cells by FCM. Predicted differential cell–cell interactions (**G**) and expression of HLA-B, HLA-A, and HLA-C (**H–J**) between patients with high (*N* = 3) and low (*N* = 3) levels of T-cell exhaustion, using scRNA-seq data. Box plots display the median (center line; also represented in the volcano plot), the 25th and 75th percentiles (box limits), and whiskers extending to the most extreme data points within 1.5× the IQR. Statistical comparisons were performed using the Wilcoxon rank-sum test with Benjamini–Hochberg correction for multiple testing. n.s., not significant; **, *P* < 0.05.

The analysis revealed that nearly all paired EMM samples (3/4) exhibited reduced cytotoxicity scores (calculated using the expression of 27 genes as previously described (35), “Methods” section “T-cell Cytotoxicity Score”) compared with their corresponding BM counterparts (Fig. 3A; Supplementary Fig. S6A). However, these comparisons were not statistically significant, perhaps due to the limited number of samples. Importantly, half of the paired EMM samples (2/4) displayed markedly elevated T-cell exhaustion scores compared with the corresponding BM samples (Fig. 3B; Supplementary Fig. S6B). Additionally, high exhaustion scores showed a very strong negative correlation with reduced CD8^+^ T-cell cytotoxic capacity in EMM samples (R = −0.86, *P* = 0.02; Fig. 3C). These observations might be further underlined by the distinct distribution of CD8^+^ T-cell subsets within the EMM samples compared with BM samples, which show high variability in the level of cytotoxicity/exhaustion (Fig. 3D and E). The most exhausted CD8^+^ T-cell subset, identified as CD8_exhausted-like TOX, was observed almost exclusively in EMM samples [median proportions of 5.8% in EMM vs. 0% in EMM_BM (*P* = 0.01) and 0.5% in RRMM_BM (*P* = 0.05) of total CD8^+^ T cells]. In contrast, clusters with the highest cytotoxicity scores were significantly less prevalent in EMM compared with EMM_BM (CD8_effector_CX3CR1; median proportion of 16.2% vs. 83.4% of total CD8^+^ T cells; *P* = 0.03) and RRMM_BM (CD8_EM_GZMK; median proportion of 6% vs. 41.8% of total CD8^+^ T cells; *P* = 0.01).

Similarly, FCM analysis revealed higher PD-1 expression on T cells from EMM samples [median proportions of 39.4% vs. 15.8% in EMM_BM (*P* = 0.05) and 25.5% in RRMM_BM (*P* = 0.05) of total T cells, Fig. 3F; Supplementary Table S8]. Additionally, when applying the traditional classification of CD8^+^ T cells into naïve, central memory (CM), effector memory (EM), and terminal EM RA-positive (TEMRA) subsets (36), we observed a progressive decrease in the percentage of naïve CD8^+^ T cells across RRMM_BM, EMM_BM, and EMM samples (median proportions of 3%, 1.1%, and 0.1% of total T cells, respectively; Supplementary Fig. S7A and S7B; Supplementary Table S9). A comparable trend was observed among CD4^+^ T cells, in which FCM analysis also showed a gradual reduction in the proportion of naïve cells across RRMM_BM, EMM_BM, and EMM samples (median proportion of 8.2%, 2.1%, and 0.08% of total T cells, respectively; Supplementary Fig. S7A and S7C).

### T-cell Exhaustion in EMM Tumors Seems to Be Driven by Direct Cell–Cell Interactions

To explore potential drivers of the high variability in T-cell exhaustion scores in the EMM cohort (Fig. 3B), we used scRNA-seq data and performed a differential cell–cell interaction analysis between EMM and T and NK cells from patients with high versus low exhaustion scores (3 vs. 3 in a pseudo-bulk approach). This analysis revealed a stronger *HLA-B*:*CD8A* interaction in the high-exhaustion group (Fig. 3G), driven by significantly higher expression of *HLA-B* (pseudo-bulk differential gene expression using DESeq2; *P* < 0.001; *adjusted P* = 0.06; Fig. 3H). A subsequent comparison (Wilcoxon test) also showed significantly higher *HLA-A* (*P* < 0.01; Fig. 3I) and *HLA-C* (*P* < 0.01; Fig. 3J) expression in the high-exhaustion group as well.

Notably, the CD8_exhausted-like TOX cluster displayed a stronger gene expression signature associated with neoantigen-reactive T cells, described by Lowery and colleagues (37), compared with other T-cell subsets (Fig. 3E). Together, the increased HLA gene expression on EMM cells in samples with high T-cell exhaustion, along with the strong neoantigen-reactive signature identified in the exhausted T cells, suggests that T-cell exhaustion in EMM may be driven by direct interactions with tumor PCs.

### Increased Presence of Regulatory CD16 Negative NK Cells in the EMM Tumor Microenvironment

**Figure 4.**
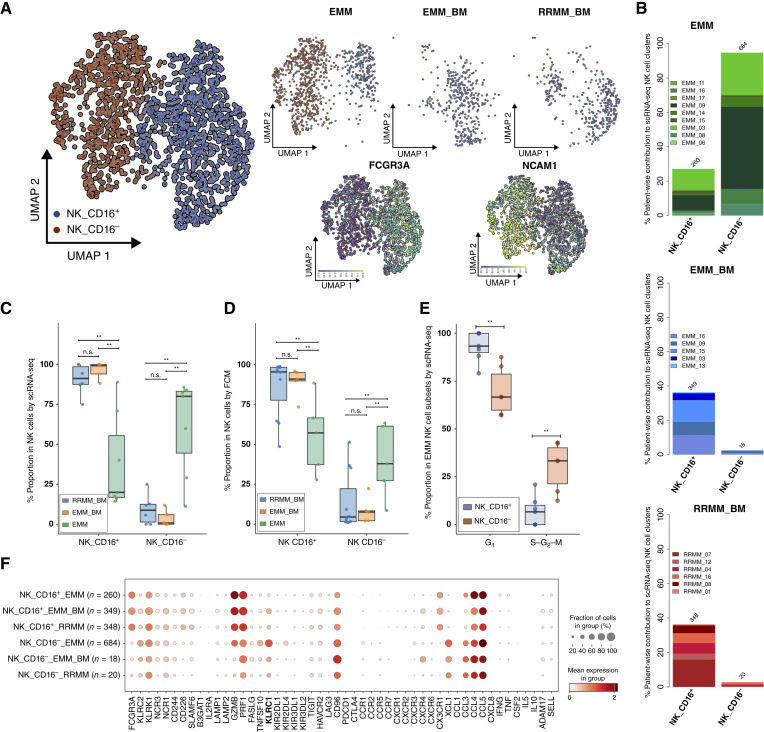
EMM tumors display high proportions of CD16^−^ NK cells. **A,** scRNA-seq data. UMAP showing clusters in the NK cell compartment, a total of 1,679 cells distinguishable by the color-coded legend (left). UMAP of NK subclusters separated for each sample type displaying cells in EMM, EMM_BM, and RRMM_BM, with the colors representing the same cell type as in the global UMAP of the NK compartment (right). UMAPs showing the expression of CD16 and CD56 in the NK cell compartment (bottom). **B,** Stacked bar plot showing patient-wise contribution to scRNA-seq NK cell clusters. The proportions sum to 100% across EMM, EMM_BM, and RRMM samples. Box plot indicating the proportion of NK cell subclusters in (**C**) scRNA-seq and (**D**) FCM cohorts, color-coded by sample type. **E,** Box plot indicating the proportion of cells in different cell-cycle phases in CD16^+^ and CD16^−^ NK cell clusters across sample types. **F,** Dot plot showing the expression of a curated list of genes in NK subclusters across different sample types. Box plots display the median (center line), the 25th and 75th percentiles (box limits), and whiskers extending to the most extreme data points within 1.5× the IQR. Statistical comparisons were performed using the Wilcoxon rank-sum test with Benjamini–Hochberg correction for multiple testing. n.s., not significant; **, *P* < 0.05.

As noted before, we dissected the NK cell compartment into two classical NK cell subpopulations, regulatory CD16^−^ and cytotoxic CD16^+^ (38), which exhibited higher and lower expression of *NCAM1* (*CD56*), respectively (Fig. 4A). When analyzing the distribution of these subsets, EMM samples were characterized by a significantly higher presence of CD16^−^ NK cells compared with BM samples [median proportions of 80% vs. 0.8% (*P* = 0.02) and 8.8% (*P* = 0.02) of total NK cells in EMM_BM and RRMM_BM, respectively; Fig. 4B and C]. Consistent trends were also observed using FCM (Fig. 4D; Supplementary Table S8).

Interestingly, the CD16^−^ cluster, which was almost exclusive to EMM, exhibited a significantly higher proportion of cells in the S–G_2_–M phase of the cell cycle compared with the CD16^+^ cluster (median proportions of 33.3% vs. 6.7% of total NK cells in EMM samples; *P* = 0.01; Fig. 4E), indicating active proliferation. Additionally, CD16^−^ NK cells from EMM samples demonstrated a significantly elevated expression of the inhibitory receptor *KLRC1* (*NKG2A*; *P* = 0.01; Fig. 4F; Supplementary Fig. S8A), which acts as a checkpoint on both NK cells and T cells (as we also observed, with the highest expression in the CD8_exhausted-like TOX cluster; Supplementary Fig. S8B) and plays a regulatory role in direct NK cell interactions with *HLA-E* on tumor cells (expressed in EMM; ref. 39).

**Figure 5.**
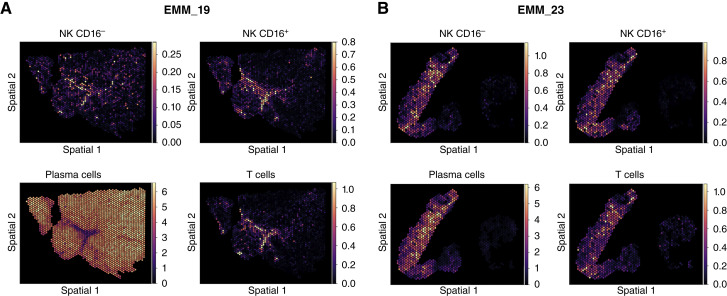
ST indicates comparable distribution of CD16^+^ and CD16^−^ NK cells across EMM samples. **A** and **B,** Estimated spatial cell type abundance of CD16^+^ and CD16^−^ NK cells, PCs, and T cells in EMM_19 (left) and EMM_23 (right) samples by cell2location. The color scale represents the estimated relative abundance of each cell type in the corresponding spatial spot: Yellow shows higher abundance of the cell type.

Importantly, we confirmed the higher abundance of CD16^−^ NK cells in EMM samples in an independent validation cohort of eight patients with EMM using ST (Supplementary Fig. S9). Moreover, using the ST approach, we demonstrated that CD16^−^ NK cells are scattered throughout the lesions without a specific pattern of accumulation (Fig. 5A and B; Supplementary Fig. S10A–S10F). However, the spatial approach lacked single-cell resolution, as each spot (55 μm in diameter) typically captures transcripts from 5 to 10 cells.

### Dissecting BM Microenvironment Differences between Patients with Multiple Myeloma with and without EMM

As the BM of patients with EMM is often almost devoid of any malignant PCs, we investigated whether immune alterations in EMM_BM contribute to or reflect PC egression. To address this, we compared BM samples from patients with EMM with those from patients with RRMM without evidence of EMM. As expected, scRNA-seq analysis revealed a considerably lower proportion of malignant PCs in EMM_BM (*N* = 5) compared with RRMM_BM (*N* = 6; median proportions of 0.2% vs. 19.5% in total nucleated cells; Fig. 1D, F, and G). Furthermore, we observed a markedly higher proportion of highly cytotoxic CD8_effector_CX3CR1 cells in EMM_BM (median proportions of 83.4% vs. 37.6% of CD8^+^ T cells; *P* = 0.08; Fig. 3D) and a significantly less EM CD8_EM_GZMK cells (median proportion of 2% vs. 41.8% of CD8^+^ T cells; *P* = 0.01; Fig. 3D). Moreover, B cells were observed to be trending toward a lower proportion in EMM_BM compared with RRMM_BM (median proportions of 0.6% vs. 1.8% of total nucleated cells; *P* = 0.8; Fig. 1D, F, and G).

Consistent with scRNA-seq findings, malignant PCs were also reduced in EMM_BM (*N* = 11) by FCM when compared with RRMM_BM (*N* = 17; median proportion of 0.5% vs. 7.1% of total nucleated cells; *P* < 0.01; Fig. 1F and G; Supplementary Fig. S11A). Likewise, B cells were less abundant in EMM_BM (median proportion of 0.5% vs. 1% of total nucleated cells; Fig. 1F and G; Supplementary Fig. S11A), alongside significantly lower levels of naïve CD8^+^ (median proportion of 1.1% vs. 3% of total T cells; *P* = 0.04; Supplementary Fig. S7A and S7B) and naïve CD4^+^ T cells (median proportion of 2.1% vs. 8.2% of total T cells; *P* = 0.04; Supplementary Fig. S7A and S7C). Conversely, CD8^+^ EM T cells showed a significantly higher proportion in EMM_BM (median proportion of 35.1% vs. 15% of total T cells; *P* = 0.04; Supplementary Fig. S7A). Finally, within the myeloid compartment, we observed no significant differences between EMM_BM and RRMM_BM using either scRNA-seq or FCM (Supplementary Fig. S11B and S11C).

In summary, we identified several potentially important differences between EMM_BM and RRMM_BM samples; however, these should be interpreted with caution, as they may simply reflect the low level of tumor PC infiltration in the BM of patients with EMM.

## Discussion

**Figure 6.**
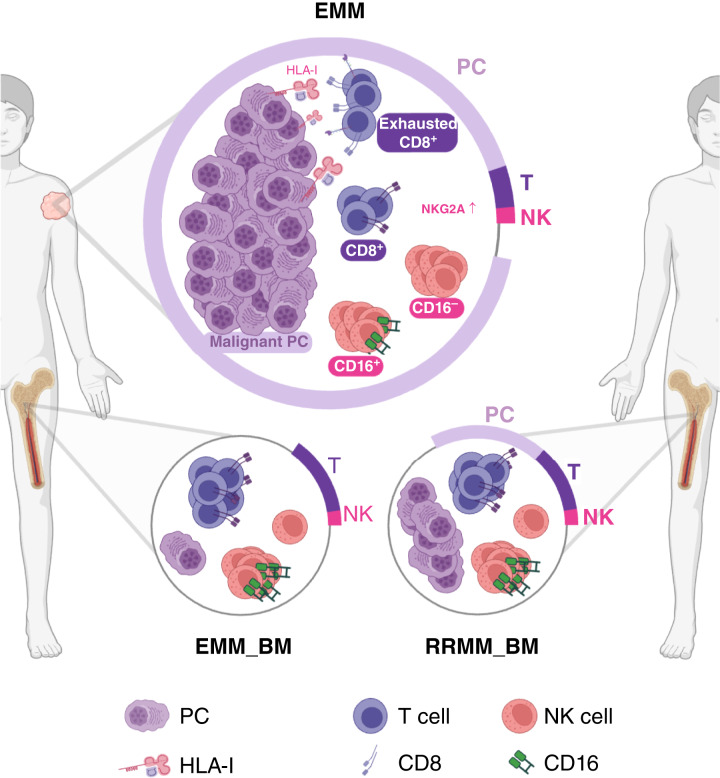
Schematic overview of our key findings on the EMM TME. EMM lesions are dominantly composed of clonal PCs. Remaining immune cell subsets are represented mainly by T and NK cells. The EMM TME is characterized by a significantly reduced E:T ratio, a higher proportion of exhausted tumor-reactive CD8^+^ T cells, and a higher proportion of less cytotoxic CD16^−^ NK cells with elevated *KLRC1* (*NKG2A*) expression. Additionally, EMM_BM shows only minimal infiltration by tumor cells. [Created in BioRender. Sevcikova, T. (2026) https://BioRender.com/n46u600]

The role of the TME in EMM remains poorly understood, largely due to the lack of data describing the presence, composition, and functional state of immune cell subsets. To the best of our knowledge, only two prior studies have provided insight into the EMM TME using single-cell techniques (18, 19). However, both of these studies analyzed a limited number of samples and did not perform a direct comparison between the TME of EMM and BM samples. Here, we present a comprehensive single-cell study of EMM tumors, leveraging paired samples (EMM and EMM_BM) and multiple single-cell techniques. Our results show that the EMM TME is characterized by a low effector-to-tumor ratio, accompanied by a higher proportion of exhausted CD8^+^ T cells with limited cytotoxicity and a high proportion of regulatory CD16^−^ NK cells compared with BM (Fig. 6).

Numerous studies consistently report significantly shorter survival for patients with multiple myeloma with EMM (compared with those without EMM) treated with various therapies, including modern immunotherapy (4–14). This may be attributed to the aggressive behavior/biology of EMM cells and thus their resistance to treatment. Our scRNA-seq data demonstrate that despite the absence of a supportive BM microenvironment, EMM cells exhibit higher levels of proliferation compared with BMPCs, consistent with previous findings obtained by IHC and bulk RNA-seq of EMM cells and by scRNA-seq of pleural effusions from patients with EMM (16–18). Conversely, we confirmed significantly lower or nearly absent infiltration of malignant PCs in the EMM_BM compared with RRMM_BM samples, consistent with our earlier findings (18). The resistance of EMM has been associated with higher genomic instability compared with multiple myeloma restricted to BM, enabling EMM cells to rapidly adapt to new challenges via clonal evolution (18–20), decreased expression of several therapeutic targets (e.g., CD38 and GPRC5D) and HLA genes (18), and, finally, with an exhausted state of tumor-infiltrating CD8^+^ T cells (12, 19).

Previously, we identified CD8^+^ T cells and NK cells as the most abundant effector subsets in EMM across all scRNA-seq samples (*N* = 5; ref. 18). However, IHC analysis revealed that some EMM tumors are depleted of T cells (2/9 patients; ref. 19), and our broader scRNA-seq/FCM sampling uncovered tumors with minimal or no apparent infiltration of immune cells. Moreover, despite the presence of effector cells in the majority of EMM tumors, the E:T ratio was significantly lower compared with BM or unrelated RRMM_BM samples. Given that a lower E:T ratio is associated with worse efficacy of both naked mAbs (40) and bsAbs (41–43), this depletion of immune effector cells in EMM may contribute to poorer treatment responses in patients with EMM.

Additionally, previous studies have suggested that the functional status of cytotoxic CD8^+^ T cells in the EMM TME may be impaired due to exhaustion (12, 19). However, similar findings have also been reported in the BM TME, in which it was demonstrated that the proportion of exhausted CD8^+^ T cells might predict patients’ progression or responses to treatment with T-cell engagers (35, 44, 45). Building on these observations, we aimed to investigate whether the functional status of CD8^+^ T cells in EMM differs from that in BM TME (EMM_BM, RRMM_BM). Our analysis revealed overall lower cytotoxicity, likely resulting from a higher exhaustion of CD8^+^ T cells in EMM, underpinned by a significant enrichment of an exhausted CD8^+^ T-cell cluster previously described as CD8_exhausted-like TOX (35). Importantly, this cluster also exhibited the overexpression of the neoantigen-reactive T-cell signature and thus closely resembled the highly exhausted cluster recently described in PMD (46). Congruently, we observed a significantly higher proportion of PD-1^+^ T cells in EMM tumors compared with BM samples using FCM. This might be primarily driven by the significantly greater exposure of T cells to malignant PCs and direct cell–cell interactions consistent with the recent work showing exhausted T cells colocalizing with malignant PCs in EMM tumors (19). Our findings further support this mechanism, as EMM cells in samples with high T-cell exhaustion expressed elevated levels of HLA class I molecules, enhancing their recognition by T cells (47).

Next, we observed a substantially higher proportion of CD16^−^ NK cells in the EMM TME compared with the BM TME. CD16^−^ NK cells are generally considered to predominantly possess regulatory functions (in contrast to cytotoxic CD16^+^ NK cells; ref. 38) and are typically enriched in solid tumors (48), and their enrichment was also described in PMD (46). Using scRNA-seq, we revealed that approximately 30% of these cells are in the G_2_–M- or S-phase, suggesting their local expansion. Importantly, it was recently shown that a higher proportion of CD16^−^ NK cells in BM is associated with worse survival in patients with multiple myeloma, independent of age, sex, ISS stage, and cytogenetic risk (30) and may be especially prognostic in daratumumab-treated patients as a reduced proportion of CD16^+^ NK cells was associated with a reduced probability of achieving minimal residual disease negativity (32), further explaining the observed limited efficacy of daratumumab in EMM (5). In addition, we observed markedly higher expression of the inhibitory receptor *KLRC1* (*NKG2A*) in these cells, which might further diminish their antitumor activity. The expression of *KLRC1* is not exclusive to NK cells and was previously identified also in CD8^+^ T cells from the tumor bed of various cancers (39). Indeed, we observed the expression of this gene also in T cells, with the highest level in the CD8_exhausted-like TOX cluster. Importantly, it was shown that *KLRC1* blockade enhances antitumor immunity mediated by both CD8^+^ T and NK cells as demonstrated *in vivo* in mice engrafted with B-cell lymphoma cells and *in vitro* using chronic myeloid leukemia cells, suggesting that blocking antibodies such as monalizumab might be a promising therapeutic approach in EMM (39).

John and colleagues (19) suggested that treatment strategies for patients with EMM should emphasize the use of immune checkpoint inhibitors. This suggestion is based on their observation of high levels of exhausted T cells in the EMM TME. Our data support this direction, demonstrating that the level of exhausted CD8^+^ T cells in EMM tumors is higher compared with their BM counterparts, potentially contributing to their limited cytotoxicity. In this context, we hypothesize that the previously proposed combined blockade of the PD-1 and KLRC1 axis (39) or anti-TIGIT and anti–LAG-3 (49) might be a potent strategy to reactivate both CD8^+^ T cells and NK cells in EMM tumors. However, the overall tumor infiltration by immune effector cells seems to be very low. Therefore, effective tumor targeting by effector cells is crucial. The RedirecTT-1 study, which combined anti-BCMA and anti-GPRC5D therapy, provided promising results in this regard, achieving a response rate of 61% in patients with EMM, with an 82% likelihood of maintaining this response after 18 months (6). In conclusion, using various single-cell techniques, we observed a profoundly lower proportion of T and NK cells relative to malignant PCs in EMM compared with BM. A detailed dissection of these subsets revealed a high proportion of regulatory CD16^−^ NK cells, resembling the situation in solid tumors (48), and a substantially higher proportion of exhausted CD8^+^ T cells in a subset of EMM samples, potentially resulting from direct tumor–T cell interactions. Moreover, we identified higher expression of various checkpoints on EMM CD16^−^ NK and CD8^+^ T cells. Despite the limited number of samples and the absence of functional experiments, which are warranted for future analyses, our findings advance the understanding of the EMM TME.

## Methods

### Patients and Data Collection

Samples were collected between 2018 and 2024 at the Department of Hematooncology, University Hospital Ostrava: 13 secondary EMM tumors; 12 BM samples from the time of EMM relapse (EMM_BM), of which 9 were paired samples; and 26 unrelated RRMM_BM samples without evidence of EMM. All samples were processed fresh after surgery (EMM) or BM aspiration (EMM_BM and RRMM_BM) and subjected to scRNA-seq library preparation and/or FCM analysis. Patients were treated in a real-world setting according to institutional guidelines. EMM tumor biopsies were performed solely when clinically indicated to confirm the EMM diagnosis. The clinical characteristics and treatment summaries of patients with EMM and RRMM are provided in Supplementary Tables S1–S3. The study was conducted in accordance with the principles of the Declaration of Helsinki and was approved by the institutional ethics committee under number 511/2022. All samples were collected upon the signed written informed consent form. Two scRNA-seq EMM samples (EMM_1 and EMM_8) and eight samples used for ST analysis were obtained from the University Hospital Würzburg (Germany) and were described previously (19).

### Cytologic Analysis of Bone Marrow Plasma Cells

BM smear slides, stained with May–Grünwald–Giemsa according to the Pappenheim method, were evaluated by a laboratory expert. At low magnification, cellularity was assessed, contamination with peripheral blood was excluded, and the number of megakaryocytes was evaluated. Plasma cells were assessed at high magnification using oil immersion as part of the differential count of nucleated cells (counted on a total of 500 nucleated cells), followed by a standard description of the individual lineages, the number of blasts, and other relevant features.

### LDH Measurement

The quantitative determination of LDH activity was measured by the Atellica CH Lactate Dehydrogenase L-P assay (cat. #1197594) using the Atellica CI Analyzer from Siemens. Measurements were taken from the serum of all patients at diagnosis and at the time of EMM or relapse without EMM (Supplementary Table S1). This excludes the external dataset used for the spatial analysis. LDH information at diagnosis is missing for patients EMM_02, EMM_13, RRMM_02, RRMM_15, and RRMM_26, as they were originally diagnosed at another center, and this information was not shared with us.

### Sample Processing

EMM samples (*N* = 13) were collected in tubes containing normal saline, processed immediately after surgery, mechanically disintegrated, and filtered through a 100 μm cell strainer (VWR International s.r.o., cat. #732-2759). BM aspirates from patients with EMM (*N* = 12) and those with RRMM (*N* = 26) were collected in ethylenediaminetetraacetic acid (EDTA)–containing tubes (SARSTEDT AG & Co., cat. #01.1605.001) and processed within 24 hours. Filtered cell suspensions from EMM and BM samples were all subjected to erythrocyte lysis using a 1× NH_4_Cl-based lysing solution [10× stock solution: NH_4_Cl 53.49 g/mol (VWR International s.r.o., cat. #21236.267), KHCO_3_ 100.12 g/mol (VWR International s.r.o., cat. #0889-1KG), EDTA solution 0.5 mol/L pH 8 (Sigma-Aldrich s.r.o., cat. #E9884; pH = 7.4)] for 15 minutes at room temperature (RT) while inverting, followed by two washes in phosphate-buffered saline (PBS, Sigma-Aldrich s.r.o., cat. #P5119) at RT.

### scRNA-Seq

Cells suspension from the samples from University Hospital Ostrava: eight EMM tumors, five EMM_BM, and six RRMM_BM were stained with 7-aminoactinomycin D (7-AAD; EXBIO, cat. #EXB0026; 5 μL of undiluted solution per 1 million cells) to discriminate between live and dead cells via FCM (Supplementary Fig. S12). All live cells (including malignant PCs as well as immune cells from the TME) were sorted into PBS containing 0.05% bovine serum albumin (BSA, Sigma-Aldrich s.r.o., cat. #05470) using a BD FACSAria III (BD Biosciences; RRID:SCR_016695) equipped with 405, 488, 561, and 633 nm excitation lasers, with purity exceeding 95%. Sample EMM_09 was subjected to additional staining with CD138-APC (MI15, BioLegend, cat. #356505) to distinguish PCs from other immune cell populations. Plasma cells (CD138^+^) and immune cells (CD138^−^) were then sorted at a ratio of 1:9 to achieve artificial enrichment of other immune cells. All samples were kept on ice for 15 to 20 minutes before loading (5,000–10,000 cells per sample) on the Chromium Controller instrument (10x Genomics) and processed by the Chromium Next GEM Single Cell 3′ RNA Reagent Kit version 3.1 [dual indexing; cat. #1000269, except for sample EMM_17 (single indexing; cat. #1000128); 10x Genomics; RRID:SCR_023672] following the manufacturer’s protocol. Sequencing was carried out by Macrogen (RRID:SCR_014454) Europe (the Netherlands) using an Illumina sequencer with 150 paired-end settings. Sequencing resulted in an estimated average of 6,326 cells, 65,195 mean reads per cell, and 2,366 median genes per cell across patients.

### scRNA-Seq Data Analysis: Preprocessing

Raw scRNA-seq data obtained from soft-tissue lesions of 10 patients with confirmed evidence of EMM, five BM samples from patients with EMM (four of them paired with the soft tissue samples), and six unrelated samples from patients with RRMM without evidence of EMM were preprocessed using CellRanger single-cell software (RRID:SCR_023221; version 7.1.0; ref. 50). CellRanger aligned the raw reads to the human reference genome GRCh38 and further generated a gene count matrix. Samples were further processed individually to analyze basic quality control matrices using the Scanpy (RRID:SCR_018139; ref. 51) toolkit for single-cell gene expression data. The cell quality check was performed on the basic quality control metrics, namely, the total number of counts, the number of genes, and the fraction of mitochondrial genes per barcode, and outliers were filtered out as determined by the median absolute deviation (https://github.com/BloodCancerResearchGroup/EMM_SC_BCD/tree/main/individual_samples). An additional filtering on the percentage of mitochondrial genes per barcode was performed to filter out dying cells in each sample. Three samples, namely, EMM_06, RRMM_04, and RRMM_07, were subjected to an additional filtering of low-quality cells based on log1p_n_genes_by_counts because of the poor overall quality of the cells in those samples. All the samples were subjected to ambient RNA correction utilizing the SoupX (RRID:SCR_019193; ref. 52) algorithm with a manually defined contamination fraction specific to each sample. Additionally, doublet cells were identified using the algorithm scDblFinder (RRID:SCR_022700; ref. 53), and only the cells identified as singlets were used for downstream analyses. Furthermore, we observed an unusual cluster that seems to be composed of erythroid cells, but we were unable to confirm its actual identity, and we excluded it from further analysis steps.

All the samples were merged for subsequent analyses, and the counts were normalized using the shifted logarithm method. Cells were scored for cell-cycle phases in the merged dataset using the score_genes_cell_cycle function in Scanpy, utilizing a predefined set of cell-cycle markers (54). Additionally, automated annotation of cell types was performed using the CellTypist (RRID:SCR_024893; ref. 34) algorithm with a built-in model for immune cells, Immune V2. ScVI (RRID:SCR_026673; ref. 55) integration was implemented on the dataset with the top 1,500 highly variable genes to account for the batch effects caused by different sample processing sites in cell clusters (Supplementary Fig. S13A and S13B). A new Uniform Manifold Approximation and Projection for Dimension Reduction (UMAP) embedding was calculated following the finding of nearest neighbors with the corrected representation from scVI, restricting the number of neighbors to 50. Following integration, Leiden (56) clustering was performed, and the optimal resolution was chosen so that the T/NK clusters and the myeloid clusters are well separated from the plasma cells with reference to the CellTypist automated annotation. The cluster annotations were further refined using markers from the literature, described in detail in the section below (“Methods” section “scRNA-Seq Cluster Annotation”).

Cytotoxicity scores were calculated for CD8^+^ T cells using Scanpy’s score_genes function with previously curated gene sets (35) as defined below, as well as by performing pseudo-bulk differential expression analysis with DESeq2 (RRID:SCR_015687) followed by gene set variation analysis (RRID:SCR_021058) single-sample gene set enrichment analysis (ssGSEA) (57) using the same gene sets. T-cell exhaustion (58) and neoantigen reactivity (37) scores were computed for CD8^+^ T cells using a similar approach. The gene sets used for computing scores throughout the publication are defined below:

T-cell exhaustion score:

“TIGIT,” “HAVCR2,” “CTLA4,” “PDCD1,” “LAG3,” “LAYN”

T-cell cytotoxicity score:

“FGFBP2,” “CX3CR1,” “FCGR3A,” “S1PR5,” “PLAC8,” “FGR,” “C1orf21,” “SPON2,” “CD300A,” “TGFBR3,” “PLEK,” “S1PR1,” “EFHD2,” “KLRF1,” “FAM65B,” “C1orf162,” “STK38,” “SORL1,” “FCRL6,” “TRDC,” “EMP3,” “CCND3,” “KLRB1,” “SAMD3,” “ARL4C,” “IL7R,” “GNLY”

Neoantigen reactivity score:

“KIR2DL4,” “HTRA1,” “LINC01480,” “LRRN3,” “ATP10D,” “CXCR6,” “GZMB,” “CTSW,” “LAYN,” “ENTPD1,” “TOX”

### scRNA-Seq Cluster Annotation

ScVI integrated data were subjected to Leiden clustering. An optimal clustering resolution was chosen to isolate the T/NK cell cluster, guided by automated cell type predictions from CellTypist. Plasma cells formed distinct clusters in nearly all samples and were annotated based on Leiden clustering results and CellTypist predictions. Additionally, copy-number analysis was used to assess the normal versus tumor status of the PCs, described in detail in the section below (“Methods” section “Copy-Number Analysis on scRNA-Seq Data”; Supplementary Fig. S14A–S14O).

Isolated T/NK cells were further filtered by removing samples with fewer than 25 cells in total. Subsequently, CD8^+^/CD4^+^ T cells and NK cells were separately isolated based on Leiden clustering (resolution = 0.05) in combination with CellTypist-informed automated annotation and were then clustered and annotated independently.

For annotation of the CD8+ T-cell clusters, the nomenclature was adopted to primarily align with existing literature (bioRxiv 2024.05.15.593193; ref. 35) to ensure consistency and enhance comparability. The EM CD8_EM_GZMK cluster displayed the highest expression of GZMK, accompanied by a reduced expression of the canonical cytotoxicity markers *GZMB* and *PRF1* (Fig. 2B). Conversely, the CD8_effector_CX3CR1 cluster exclusively expressed the chemokine receptor *CX3CR1* and demonstrated an elevated expression of cytotoxicity markers *PRF1* and *GNLY*. The cluster CD8_proliferating_MKI67 showed dominant expression of proliferation markers *MKI67* and *TOP2A*. Analogously, the CD8_exhausted-like TOX cluster exhibited peak expression of the exhaustion markers *HAVCR2* (TIM-3), *LAG-3*, and *TOX*. The CD8_T_Apoptotic cluster was characterized by a higher percentage of mitochondrial genes and minimal gene signatures for oxidative phosphorylation, glycolysis, and fatty acid metabolism. The CD8_TRM_like cluster, with low cytotoxicity, displayed a tissue resident–like phenotype with high expression of *CD69*, *CD44*, *ITGA1*, and *ZNF683* and no evidence of *KLF2*. The γδ T-cell cluster displayed high *TRDC* expression, whereas the NK T cell (NKT) cluster showed characteristics of both T (*CD3E*, *CD3D*, *CD3G*) and NK cells [*KLRB1*, *FCGR3A* (CD16)].

Annotation of CD4^+^ T-cell clusters was primarily guided by marker genes reported previously (35, 44, 59), identifying three distinct CD4^+^ T-cell clusters and one CD8^+^ T-cell cluster (CD8^+^ naïve T cells). CD4^+^ effector T cells displayed a higher expression of markers *GZMK*, *GZMA*, and *CCL5*, with low-to-moderate expression of *SELL* and *CCR7*, whereas the CD4^+^ naïve cells showed an elevated expression of *SELL*, *CCR7*, *TCF7*, and *LEF1*. CD8^+^ naïve cells were identified within the CD4^+^ T-cell compartment, reflecting their very low abundance and tendency to cluster more closely with naïve CD4^+^ cells rather than with CD8^+^ clusters, a pattern also reported by others (bioRxiv 2024.05.15.593193; ref. 60).

Tregs exhibited the highest expression of *TOX* as well as *IKZF2*. To further validate this annotation, we performed additional marker gene analysis using the Scanpy rank_gene_groups function (Wilcoxon rank-sum test) to identify key marker genes. This approach identified FOXP3 as the top-ranked marker by log_2_ fold change. Other key Treg markers (e.g., *CTLA4*, *TNFRSF18*, *IKZF2*, and *IL2RA*) were also found among the top markers. Furthermore, gene set enrichment analysis identified multiple Treg-associated gene sets as the top enriched pathways across several MSigDB databases (Computational Celltype, Co.Expression.Azimuth.2023, and Co.Expression.CellMarker.2024). In addition, we scored all cells using the curated gene set HE_LIM_SUN_FETAL_LUNG_C4_TREG_CELL from the MSigDB C8 database (using the Scanpy score_genes function), which again identified these cells as having markedly higher Treg identity scores compared with other cells.

NK cells segregated into two distinct clusters: a CD16^−^ NK cell cluster characterized by high *NCAM1* (CD56) expression and reduced *FCGR3A* (CD16) expression and a CD16^+^ NK cell cluster marked by elevated *FCGR3A* (CD16) expression and lower *NCAM1* (CD56) expression. In addition, NKT cells clustered alongside NK cells at the interface with CD8^+^ T-cell clusters, exhibiting features of both T cells and NK cells as described above. The UMAP of T/NK subclusters for each sample separately is provided (Supplementary Fig. S15A–S15C).

For myeloid subset annotation, we used sample-wise integration using scVI with sample type as a covariate. The initial basis for annotation was created using CellTypist’s Immune_All_Low.pkl model. This approach misclassified a substantial portion of *FCGR3B*- and *CXCR2*-expressing neutrophils as monocytes, which were manually reclassified. Additionally, megakaryocytic cells, mast cells, and fibroblasts were merged into “Other minor populations,” as they were not designated to be detectable by FCM. The annotation was verified using several markers: neutrophils (*FCGR3B*, *CXCR2*, *LCN2*, *CAMP*, *MMP9*, *MMP8*, *AZU1*, *AQP9*, *LYZ*, *MPO*, *S100A8*, *LTF*, *PRTN3*, *CEACAM8*), B cells (*CD79A*, *CD79B*, *PAX5*), fibroblasts (*ACTA2*, *FN1*, *COL5A1*, *SFRP1*, *DCN*, *COL1A1*, *COL1A2*), dendritic cells (*CD74*, *HLA-DRA*, *HLA-DPA1*, *HLA-DPB1*, *HLA-DRB1*, *HLA-DQA1*, *CLEC9A*, *XCR1*, *CADM1*, *FCER1A*, *CD1C*, *IRF4*, *LILRA4*, *GZMB*, *CLEC4C*), monocytes/macrophages (*CD14*, *FCGR3A*, *VCAN*, *C1QA*, *C1QB*), erythroid cells (*GATA1*, *HBB*, *KLF1*, *HBA1*, *HBA2*, *HBD*, *GYPA*, *ALAS2*), and megakaryocytes (*ITGA2B*, *VWF*).

Building on the main annotation, we further subclustered neutrophil, monocyte/macrophage, and dendritic cell populations to increase resolution. To select the resolution parameter for Leiden clustering, we evaluated correlation matrices and chose values that minimized the occurrence of highly similar clusters. Within the isolated neutrophil population, we observed that cluster 6 (at resolution 0.6) separated distinctly but lacked specific marker genes. At higher resolutions, subfractions of this cluster showed stronger similarity to different neighboring subclusters while being dissimilar to each other. To reflect this, we merged these cells with the most similar clusters. We also merged two clusters (0 and 1) with very high correlation. This resulted in four unique neutrophil clusters, three of which were annotated based on dominant marker gene expression, applying the following thresholds: (i) ≥85% of cells within the cluster expressed the marker gene and (ii) ≤35% of cells outside the cluster (within the neutrophil compartment) expressed the same gene. The cluster with high expression of *MKI67* was annotated as immature neutrophils. Similarly, we subclustered the monocyte/macrophage cluster, in which the cluster with markedly highest expression of *FCGR3A* (CD16) was denoted as nonclassical monocytes. The cluster with expression of *C1QA*, *C1QB*, and high expression of HLA-II molecules was annotated as macrophages. All other clusters with high expression of *VCAN*, *CD14*, *S100A9*, and *S100A8* were annotated as classical monocytes. Lastly, dendritic cells were subclustered using these markers: cDC1 (*CLEC9A*, *XCR1*, *IRF8*, *CADM1*), cDC2 (*CD1C*, *FCER1A*, *IRF4*, *CLEC10A*), and pDC (*CLEC4C*, *IL3RA*, *TCF4*, *LILRA4*, *IRF7*, *GZMB*).

The derived cell labels were then transferred to the initial dataset with batch effects from sampling sites removed, preserving patient-specific characteristics.

### Cell–Cell Communication Analysis on scRNA-Seq Data

Cell–cell communication analysis was performed on PCs, CD8^+^ T cells, and NK cells from the three samples with the highest (EMM_14, EMM_15, and EMM_16) and three samples with the lowest levels (EMM_03, EMM_09, and EMM_11) of T-cell exhaustion, as determined by pseudo-bulk scoring. CD8^+^ T cells and NK cells were defined as receiver cell types, and candidate receptors were included if they were expressed in at least 20% of cells within their respective compartments. Plasma cells were defined as the sender cell type. Ligands were required to be expressed in at least 5% of PCs and to be differentially expressed between high- and low-exhaustion groups (adjusted *P* value < 0.1, |log_2_ fold change| > 1), as determined by pseudo-bulk analysis using the decoupler package. The potential for interaction was then inferred using the NicheNet (RRID:SCR_023158; ref. 61) framework, with ligands considered candidate interactors if they were predicted to interact with expressed receptors.

In parallel, ligand–receptor interactions were inferred using the rank_aggregate function from the LIANA (62) package (Python implementation version 1.4.0). Results were filtered for significance (cellphone_pvals < 0.05) and for ligand complexes containing the previously identified candidate ligands. Interaction networks were visualized using the dotplot function from LIANA.

### Copy-Number Analysis on scRNA-Seq Data

Copy-number analysis on EMM (*n* = 10) and RRMM_BM (*n* = 5) samples with at least 10 PCs was performed using InferCNV (https://github.com/broadinstitute/infercnv, accessed August 1, 2025; RRID:SCR_021140) version 1.20.0 separately for each sample. Immune cells from corresponding samples were used as reference cells where available. For samples EMM_06 and EMM_17 with minimal immune cells, InferCNV was run with immune cells from EMM_11 as the reference.

Additionally, the code used for scRNA-seq data analysis, including detailed quality control steps and threshold settings, clustering and annotation, and the generation of publication figures, is available at https://github.com/BloodCancerResearchGroup/EMM_SC_BCD.

### FCM Analysis

For characterization of basic immune subpopulations, samples [EMM (*N* = 13), EMM_BM (*N* = 11), and RRMM_BM (*N* = 17)] were stained using the standardized EuroFlow eight-color PC disorders panel tube 1 (63). All flow cytometric stainings were carried out for 15 minutes at RT, followed by washing with PBS. The standardized eight-color EuroFlow PCD panel tube 1 was used to characterize basic immune subpopulations (T cells, B cells, NK cells, monocytes/macrophages, neutrophils, PCs, erythroid cells, and other minor populations). The number of nucleated cells analyzed with this panel represents a median of 772,622 (range: 47,681–2,500,565). The following fluorophore–antibody conjugates were used: CD38 multiepitope FITC (clone “multi epitope,” Cytognos, cat. #CYT-38F2), CD56 PE (C5.9, Cytognos, cat. #CYT-56PE), CD45 PerCP-Cy5.5 (HI30, BioLegend, cat. #304028), CD19 PE-Cy7 (J3-119, Beckman Coulter, cat. #IM3628), CD117 APC (clone 104D2, BD Biosciences, cat. #333233), CD81 APC-C750 (M38, Cytognos, cat. #CYT-81AC750), CD138 BV421 (MI15, BD Biosciences, cat. #562935), and CD27 BV510 (O323, BioLegend, cat. #302835).

For more detailed characterization of T-cell subsets, samples [EMM (*N* = 5), EMM_BM (*N* = 4), and RRMM_BM (*N* = 11)] were stained using three different eight-color tubes (64): (i) tube 1 (inspired by EuroFlow T-cell chronic lymphoproliferative disorder tubes) for dissection of T-cell subtypes CD4^+^, CD8^+^, CD4^−^CD8^−^ double negative, CD4^+^CD8^+^ double positive, CD27^+^ CD45RA^+^ naïve, CD27^+^ CD45RA^−^ CM, CD27^−^ CD45RA^−^ EM, and CD27^−^ CD45RA^+^ terminal EM T cells; (ii) tube 2 representing the EuroFlow lymphoid screening tube (LST) for the detection of TCRγδ+ T cells; and (iii) tube 3 for the detection of Tregs (CD4^+^ CD25^+^ CD127^−^). The number of lymphocytes analyzed with this panel represents a median of 15,074 (range: 675–100,174). Following fluorophore-antibody conjugates were used: CD27 FITC (L128, BD Biosciences, cat. #654664), CD197 PE (3D12, Invitrogen, cat. #12-1979-42), CD3 PerCP-Cy5.5 (SK7, BD Biosciences, cat. #340948), CD45RO PE-Cy7 (UCHL-1, BD Biosciences, cat. #649452), CD45RA APC (HI100, BD Biosciences, cat. #550855), CD8 APC-H7 (SK1, BD Biosciences, cat. #641409), CD4 PB (MEM-241, EXBIO, cat. #PB-359-T025), CD45 PO (HI30, EXBIO, cat. #PO-684-T025), CD8 FITC (SK1, BD Biosciences, cat. #340692), sIgLambda F(ab′)2 FITC (BD Biosciences, cat. #332788), CD56 PE (C5.9, Cytognos, cat. #CYT-56PE), sIgKappa PE (TB 28-2, BD Biosciences, cat. #347246), CD5 PerCP-Cy5.5 (L17F12, BD Biosciences, cat. #341099), CD19 PE-Cy7 (J3-119, Beckman Coulter, cat. #IM3628), TCRgd PE-Cy7 (11F2, BD Biosciences, cat. #655410), CD3 APC (SK7, BD Biosciences, cat. #340661), CD38 APC-H7 (HB7, BD Biosciences, cat. #656646), CD20 PB (2H7, EXBIO, cat. #PB-638-T025), CD127 BV421 (HIL-7R-M21, BD Biosciences, cat. #562436), CD25 PE (2A3, BD Biosciences, cat. #341009), and CD4 PerCP-Cy5.5 (SK3, BD Biosciences, cat. #341653).

For typization of CD56^+^ CD16^−^ and CD56^−^ CD16^+^ NK cell subsets, samples [EMM (*N* = 5), EMM_BM (*N* = 5), and RRMM_BM (*N* = 11)] were stained with two 8-color tubes. The numbers of lymphocytes analyzed with this panel was a median of 101,993.5 (range: 1,043–373,244). The following fluorophore–antibody conjugates were utilized: CD38 multiepitope FITC (clone “multi epitope,” Cytognos, cat. #CYT-38F2), CD56 PE (N901, Beckman Coulter, cat. #IM2073U), CD3 PerCP-Cy5.5 (UCHT1, Beckman Coulter, cat. #B49203), CD57 PE-Cy7 (QA17A04, BioLegend, cat. #393309), NKG2A APC (Z199, Beckman Coulter, A60797), CD16 APC-AF750 (3G8, Beckman Coulter, cat. #B49184), SLAMF7 BV421 (235614, BD Biosciences, cat. #750819), CD45 Krome Orange (J33, Beckman Coulter, cat. #B36294), PD-1 AF488 (EH12.2H7, EXBIO, cat. #1F-176-T100), NKp46 APC (9E2, Sony Biotechnology, cat. #2259585), and KIR2DL1 BV421 (HP-3E4, BD Biosciences, cat. #564318).

FCM samples were processed according to standardized EuroFlow operating procedures (65) and acquired using a FACSCanto II flow cytometer [Becton Dickinson Biosciences (BDB)] equipped with FACSDiva 6.1 software (BDB). Instrument settings and performance monitoring were conducted daily using the Cytometer Tracking and Setup Beads (BD Biosciences, cat. #656505) and rainbow eight-peak beads (SPHERO Rainbow Calibration Particles, Spherotech, cat. #RCP-30-5A). Fcs files were analyzed using commercial FCM software (Kaluza analysis 2.1, Beckman Coulter, or Infinicyt 2.0.6, Cytognos). Initial gating included the removal of debris (forward scatter area/side scatter area plot) and doublets (forward scatter area/forward scatter height), followed by gating of leukocytes or nucleated BM cells (CD45/side scatter area). The gating strategy for each of the panels is described in Supplementary Figs. S16–S18. The composition of individual panels is summarized in Supplementary Table S10. Clusters containing more than 20 events were considered a valid population for rare subtypes.

### Spatial Data Analysis

To estimate cell type abundances at each spot of the ST data, we used our EMM scRNA-seq data as a reference in combination with Cell2location (RRID:SCR_024859; ref. 19) version 0.1.3. scRNA-seq data were used to generate expression signatures through Cell2location’s negative binomial regression model, with a training mini-batch size of 3,000 and a total of 250 training epochs. Prior to analysis, mitochondrial genes were removed from the Visium (RRID:SCR_023571) dataset. For cell abundance estimation, untransformed and unnormalized mRNA counts of selected genes were considered. Genes were included if they were expressed in more than 3% of cells or had a mean expression level exceeding 1.12. The number of cells per spot was set to 15, based on DAPI staining counts.

*Number of genes detected per spot*: Across the cohort, the median number of genes per spot was estimated to be 1,208.

*Data normalization and batch correction*: We used cell2location (RRID:SCR_024859) version 0.1.3 to integrate the ST data with matching scRNA-seq data. During reference creation, we applied scVI, as implemented in cell2location, to correct for batch effects between single-cell datasets before projection onto the spatial data.

*Cell type annotation*: Cell types were inferred by projecting the single-cell reference onto the ST data using cell2location. Annotations were based on well-established marker genes and validated through expert curation.

### Statistical Analysis

All statistical analyses were performed using R version 4.3.2 (RRID:SCR_001905; www.r-project.org). Data for individual samples are presented as medians, as indicated in the text. Statistical significance was assessed using Pearson’s *χ*^2^ test or Fisher’s exact test for categorical variables and the Wilcoxon rank-sum test or Wilcoxon rank-sum exact test for continuous variables. Although the Wilcoxon rank-sum test was used throughout the publication, other tests were applied appropriately based on sample size and the presence of ties, following the default settings of the gtsummary version 1.6.23 (RRID:SCR_021319) R package. A *P* value of <0.05 was considered statistically significant.

## Supplementary Material

Table S1 Clinical characteristics

Table S2 Summary of clinical characteristics

Table S3 Treatment characteristics

Table S4 Treatment summary

Table S5 Summary of clinical characteristics of EMM patients in FC and scRNA cohort

Table S6 Summary of clinical characteristics of RRMM patients in FC and scRNA cohort

Table S7 Number of cells in each cell type identified by FCM in total nucleated cells

Table S8 Quantification of PD-1–positive cells and NK cell subsets identified by FCM

Table S9 Number of cells in each cell type identified by FCM in total T cells

Table S10 Composition of individual panels for FCM

Figure S1 Figure S1 showing correlation between cell types measured by scRNAseq and FCM

Figure S2 Comparison of scRNA-seq and FCM cohorts

Figure S3 Effector:Tumor ratio

Figure S4 Increased proliferation in EMM cells

Figure S5 T/NK compartment by scRNAseq - downsampled

Figure S6 Median cytotoxicity and exhaustion scores of CD8+ T cells

Figure S7 T/NK compartment by FCM

Figure S8 Expression of KLRC1(NKG2A)

Figure S9 NK cell abundance in spatial data

Figure S10 Cell type abundances measure by Spatial transcriptomic data

Figure S11 Myeloid subclusters measure by FCM and scRNAseq

Figure S12 Representative gating strategy of 7-AAD staining

Figure S13 Batch correction

Figure S14 Copy number analysis on EMM (N = 10) and RRMM_BM (N = 5) samples

Figure S15 UMAPs separated by samples

Figure S16 Representative gating strategy of basic immune subpopulations

Figure S17 Representative gating strategy of T cells

Figure S18 Representative gating strategy of NK cells

## Data Availability

All the newly generated scRNA-seq data are available at the European genome-phenome archive (EGA) archive under the accession number EGAD50000001511. EMM scRNA-seq samples EMM_03_1, EMM_11, EMM_14_1, EMM_14_2, EMM_16, and EMM_15_1 are available in EGA archive as part of dataset ID EGAD50000000053 under the accession numbers EGAN50000005163, EGAN50000005177, EGAN50000005164, EGAN50000005198, EGAN50000005163, and EGAN50000005213, respectively (18). These sample IDs are visible as metadata once access is granted. ST data are available in the EGA archive under the accession number EGAS50000000227 (19).
