# Supplementary material for: Composition and Functional State of T and NK Cells in the Extramedullary Myeloma Tumor Microenvironment
Source: Blood Cancer Discov. 2025 Nov 14;7(2):250–65. doi: 10.1158/2643-3230.BCD-25-0170 (PMC13012251; doi:10.1158/2643-3230.BCD-25-0170)
Supplement: Figure S1 — showing correlation between cell types measured by scRNAseq and FCM [file bcd-25-0170_figure_s1_suppsf1.pdf]

## Supplementary Figure 1

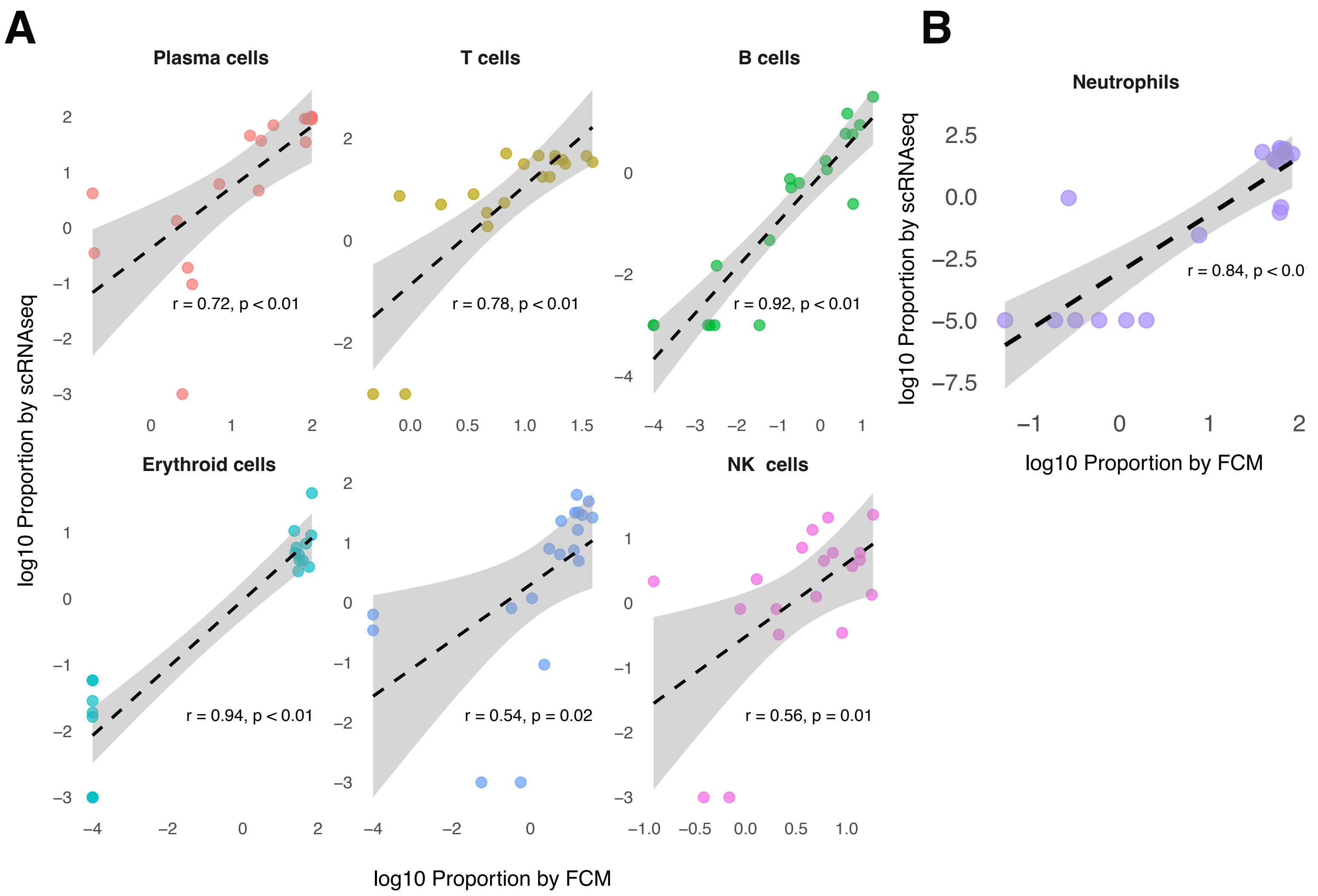


**Supplementary Figure 1:** Correlation between cell types measured by scRNAseq and FCM : **(A)** Pearson correlation of proportion of celltypes by scRNAseq and FCM excluding neutrophils **(B)** Correlation of Neutrophil proportions by scRNAseq and FCM
