# Supplementary material for: Composition and Functional State of T and NK Cells in the Extramedullary Myeloma Tumor Microenvironment
Source: Blood Cancer Discov. 2025 Nov 14;7(2):250–65. doi: 10.1158/2643-3230.BCD-25-0170 (PMC13012251; doi:10.1158/2643-3230.BCD-25-0170)
Supplement: Figure S2 — Comparison of scRNA-seq and FCM cohorts [file bcd-25-0170_figure_s2_suppsf2.pdf]

Supplementary Figure 2

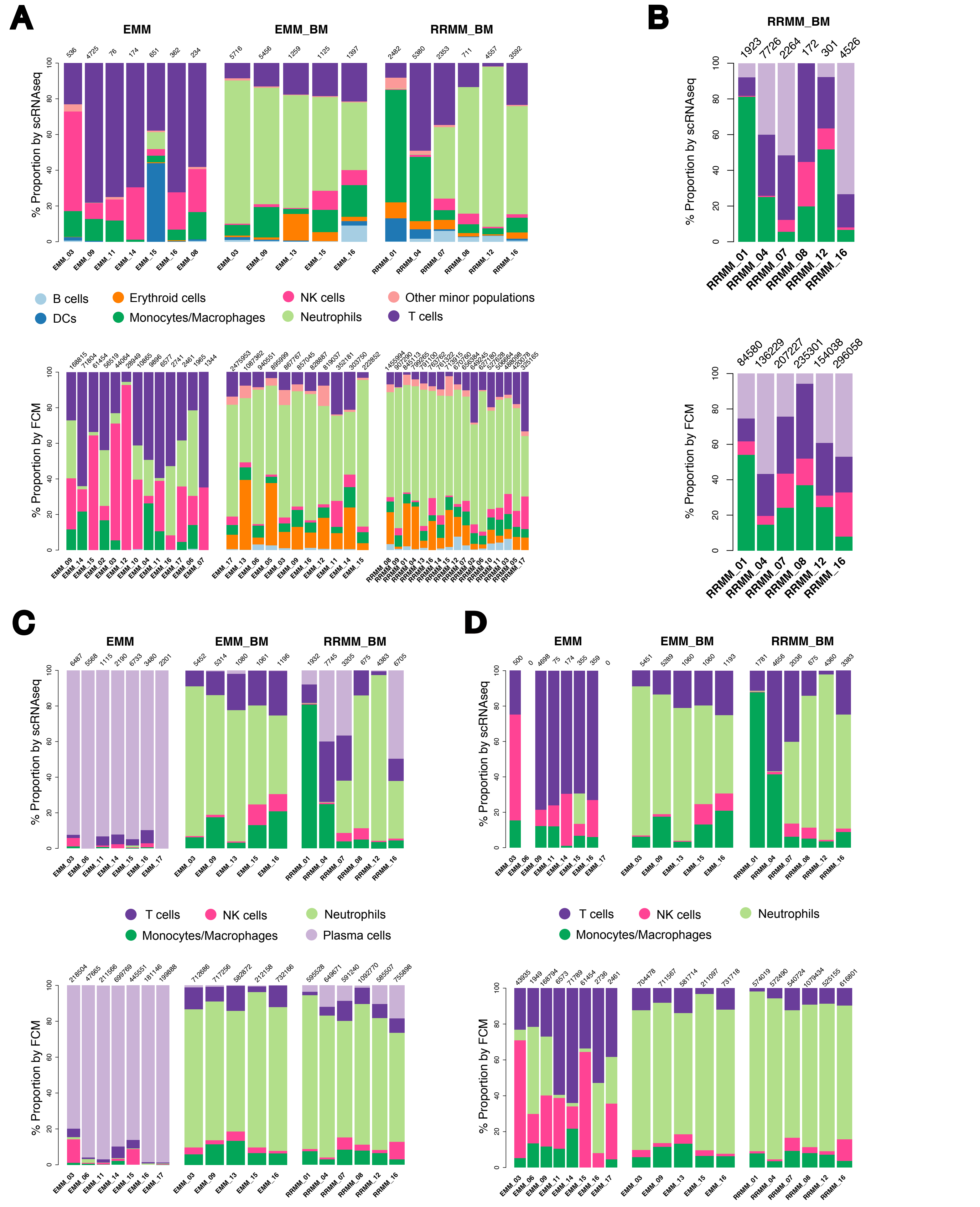

Supplementary Figure 2: Comparison of scRNA-seq and FCM cohorts: Stacked barplot indicating proportion of **(A)** different cell types excluding PCs in each sample, **(B)** T, NK, PCs and monocyte/macrophages in RRMM_BM samples. Direct comparison of cell type proportions: T, NK, monocyte/macrophages and neutrophils **(C)** including and **(D)** excluding PCs grouped by sample types by **(top)** scRNAseq and **(bottom)** FCM cohorts.
