# Supplementary material for: Composition and Functional State of T and NK Cells in the Extramedullary Myeloma Tumor Microenvironment
Source: Blood Cancer Discov. 2025 Nov 14;7(2):250–65. doi: 10.1158/2643-3230.BCD-25-0170 (PMC13012251; doi:10.1158/2643-3230.BCD-25-0170)
Supplement: Figure S3 — Effector:Tumor ratio [file bcd-25-0170_figure_s3_suppsf3.pdf]

Supplementary Figure 3

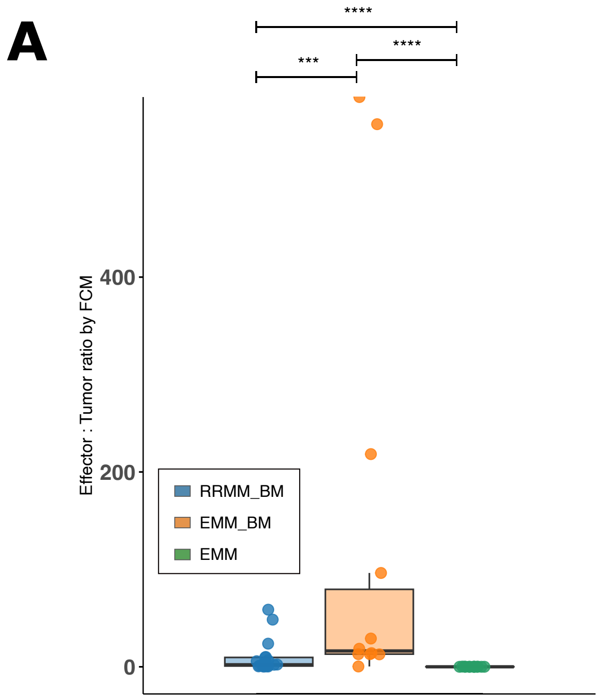

Supplementary Figure 3: Effector:Tumor ratio **(A)** Box plot indicating effector : Tumor ratio in EMM, EM_BM and RRMM_BM samples. Boxplots display the median (center line), the 25th and 75th percentiles (box limits), and whiskers extending to the most extreme data points within 1.5× the interquartile range. Statistical comparisons were performed using Wilcoxon rank-sum test with Benjamini–Hochberg correction for multiple testing. **p < 0.05; ***p < 0.01; ****p < 0.001
