# Supplementary material for: Composition and Functional State of T and NK Cells in the Extramedullary Myeloma Tumor Microenvironment
Source: Blood Cancer Discov. 2025 Nov 14;7(2):250–65. doi: 10.1158/2643-3230.BCD-25-0170 (PMC13012251; doi:10.1158/2643-3230.BCD-25-0170)
Supplement: Figure S4 — Increased proliferation in EMM cells [file bcd-25-0170_figure_s4_suppsf4.pdf]

Supplementary Figure 4

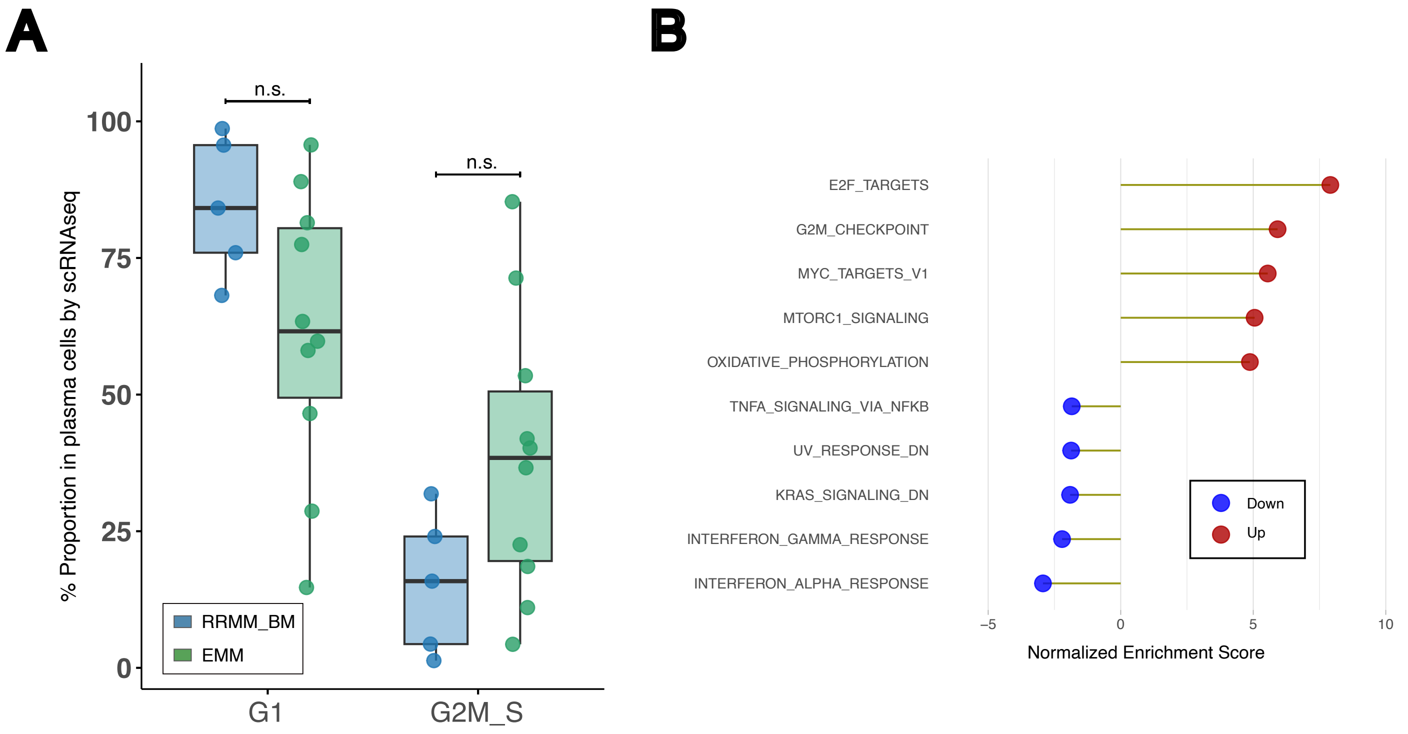

Supplementary Figure 4: Increased proliferation in EMM cells **(A)** Box plot indicating proportion of cells in G1 and G2M/S phases of cell cycle in PCs (EMM vs RRMM_BM) estimated by scRNAseq **(B)** Significantly enriched Hallmark pathways from pseudobulk differential expression analysis of PCs in EMM vs RRMM_BM by scRNAseq. Boxplots display the median (center line), the 25th and 75th percentiles (box limits), and whiskers extending to the most extreme data points within 1.5× the interquartile range. Statistical comparisons were performed using Wilcoxon rank-sum test with Benjamini–Hochberg correction for multiple testing. n.s = not significant, **p < 0.05; ***p < 0.01; ****p < 0.001
