# Supplementary material for: Composition and Functional State of T and NK Cells in the Extramedullary Myeloma Tumor Microenvironment
Source: Blood Cancer Discov. 2025 Nov 14;7(2):250–65. doi: 10.1158/2643-3230.BCD-25-0170 (PMC13012251; doi:10.1158/2643-3230.BCD-25-0170)
Supplement: Figure S5 — T/NK compartment by scRNAseq - downsampled [file bcd-25-0170_figure_s5_suppsf5.pdf]

Supplementary Figure 5

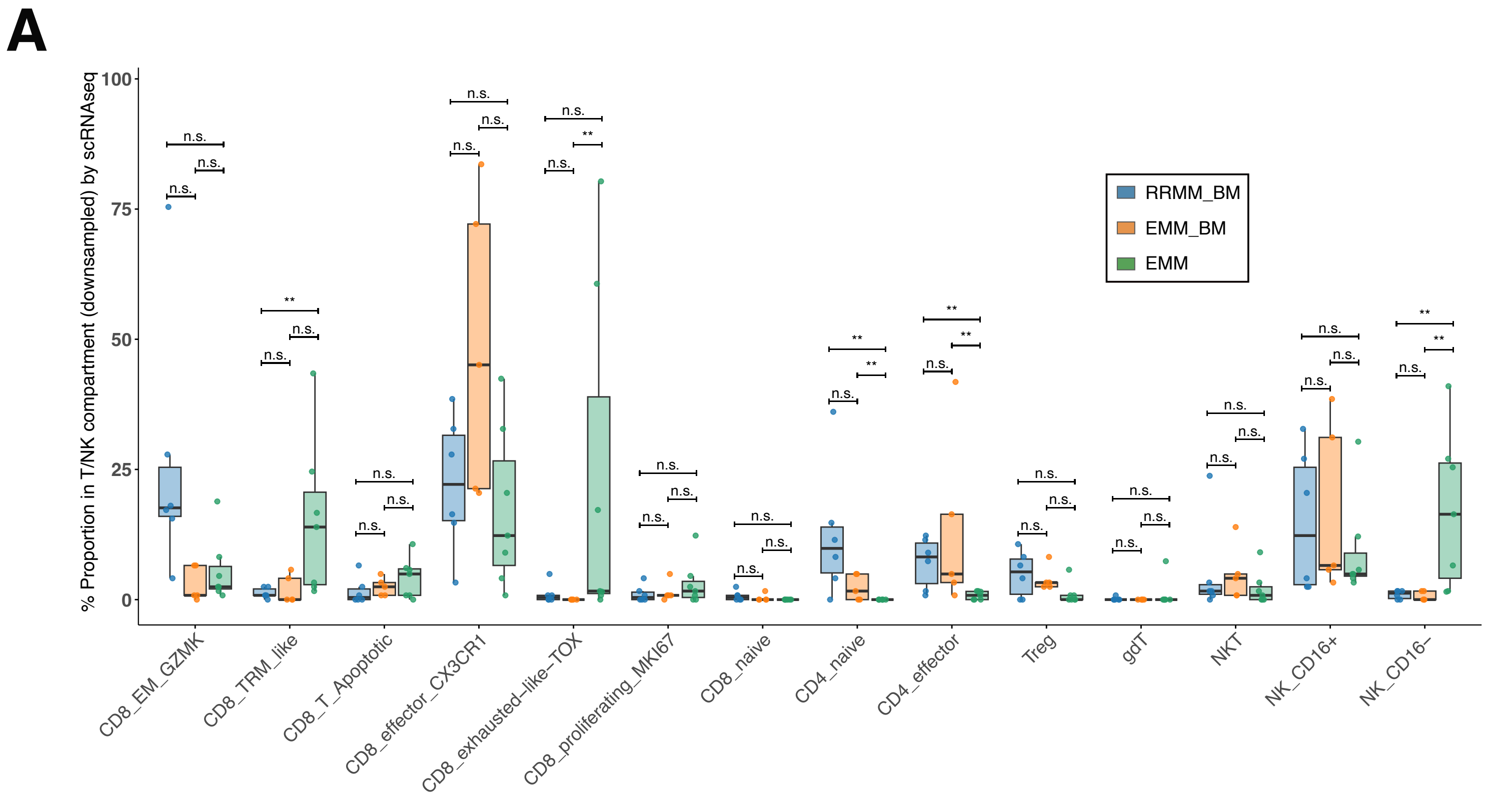

Supplementary Figure 5: T/NK compartment by scRNAseq - downsampled **(A)** Boxplot showing proportion of subclusters in down sampled T/NK compartment. All samples were downsampled to 122 immune cells, except for EMM_11 that had 66 immune cells in total. Boxplots display the median (center line), the 25th and 75th percentiles (box limits), and whiskers extending to the most extreme data points within 1.5× the interquartile range. Statistical comparisons were performed using Wilcoxon rank-sum test with Benjamini–Hochberg correction for multiple testing. n.s. = not significant**p < 0.05; ***p < 0.01; ****p < 0.001
