# Supplementary material for: Composition and Functional State of T and NK Cells in the Extramedullary Myeloma Tumor Microenvironment
Source: Blood Cancer Discov. 2025 Nov 14;7(2):250–65. doi: 10.1158/2643-3230.BCD-25-0170 (PMC13012251; doi:10.1158/2643-3230.BCD-25-0170)
Supplement: Figure S6 — Median cytotoxicity and exhaustion scores of CD8+ T cells [file bcd-25-0170_figure_s6_suppsf6.pdf]

## Supplementary Figure 6

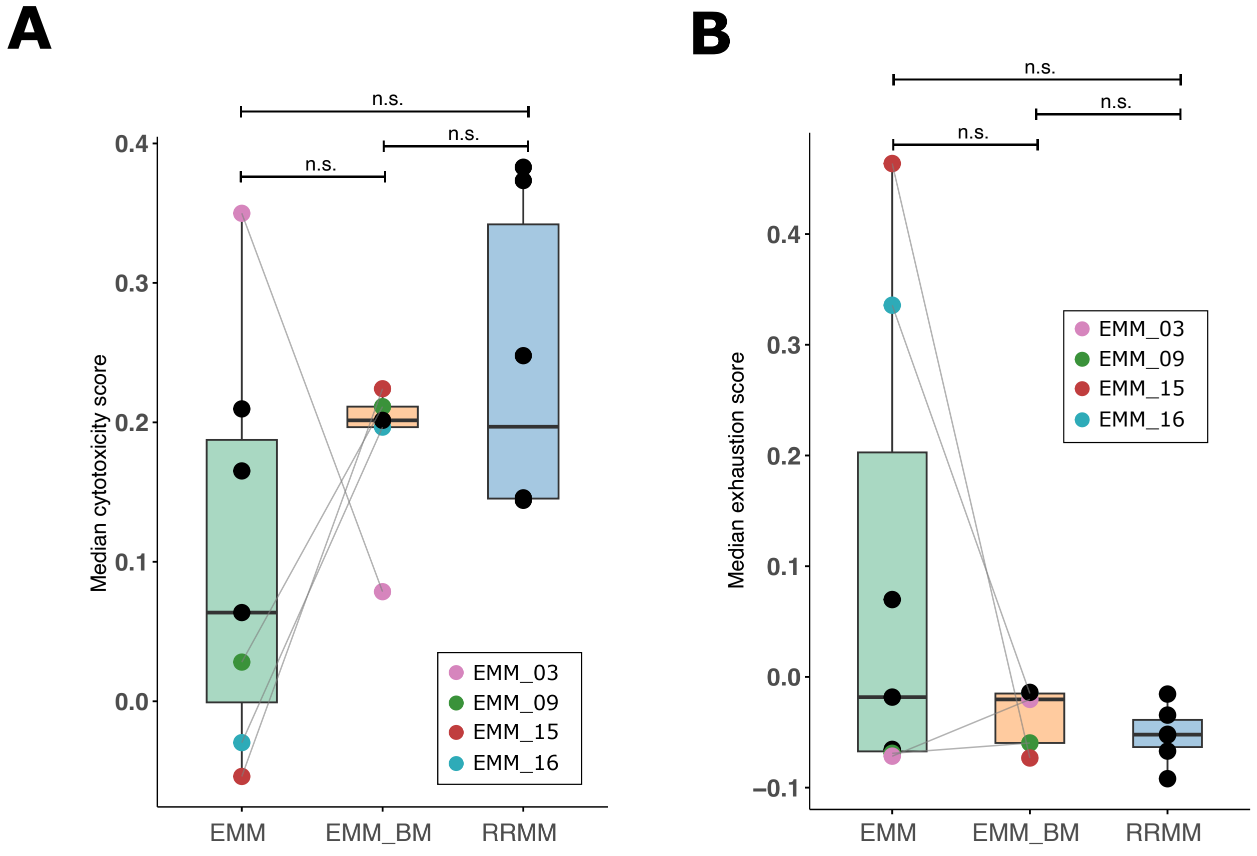


**Supplementary Figure 6:** Median cytotoxicity and exhaustion scores of CD8⁺ T cells **:** Boxplot comparing median of **(A)** cytotoxicity score and **(B)** exhaustion score by scRNAseq. Boxplots display the median (center line), the 25th and 75th percentiles (box limits), and whiskers extending to the most extreme data points within 1.5× the interquartile range. Statistical comparisons were performed using Wilcoxon rank-sum test with Benjamini–Hochberg correction for multiple testing. n.s. = not significant, **$p < 0.05$; ***$p < 0.01$; ****$p < 0.001$
