# Supplementary material for: Composition and Functional State of T and NK Cells in the Extramedullary Myeloma Tumor Microenvironment
Source: Blood Cancer Discov. 2025 Nov 14;7(2):250–65. doi: 10.1158/2643-3230.BCD-25-0170 (PMC13012251; doi:10.1158/2643-3230.BCD-25-0170)
Supplement: Figure S7 — T/NK compartment by FCM [file bcd-25-0170_figure_s7_suppsf7.pdf]

Supplementary Figure 7

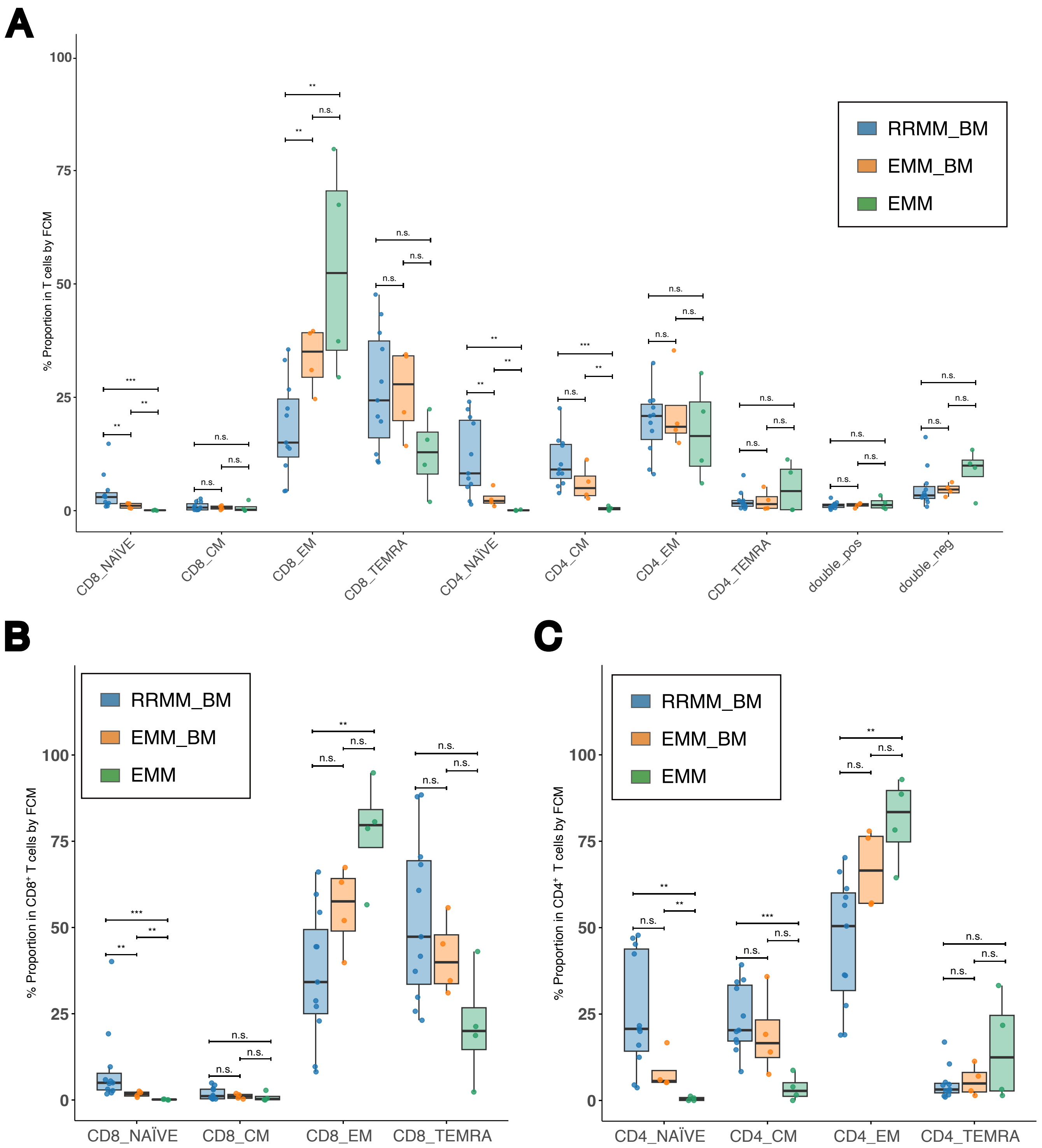

Supplementary Figure 7: T/NK compartment by FCM: Boxplots showing the proportion of T cell subclusters in of **(A)** all of T cells, **(B)** CD8+ T cells, **(C)** CD4+ T cells by FCM. Boxplots display the median (center line), the 25th and 75th percentiles (box limits), and whiskers extending to the most extreme data points within 1.5× the interquartile range. Statistical comparisons were performed using Wilcoxon rank-sum test with Benjamini–Hochberg correction for multiple testing. n.s. = not significant, $**p < 0.05$; $***p < 0.01$; $****p < 0.001$
