# Supplementary material for: Composition and Functional State of T and NK Cells in the Extramedullary Myeloma Tumor Microenvironment
Source: Blood Cancer Discov. 2025 Nov 14;7(2):250–65. doi: 10.1158/2643-3230.BCD-25-0170 (PMC13012251; doi:10.1158/2643-3230.BCD-25-0170)
Supplement: Figure S8 — Expression of KLRC1(NKG2A) [file bcd-25-0170_figure_s8_suppsf8.pdf]

Supplementary Figure 8

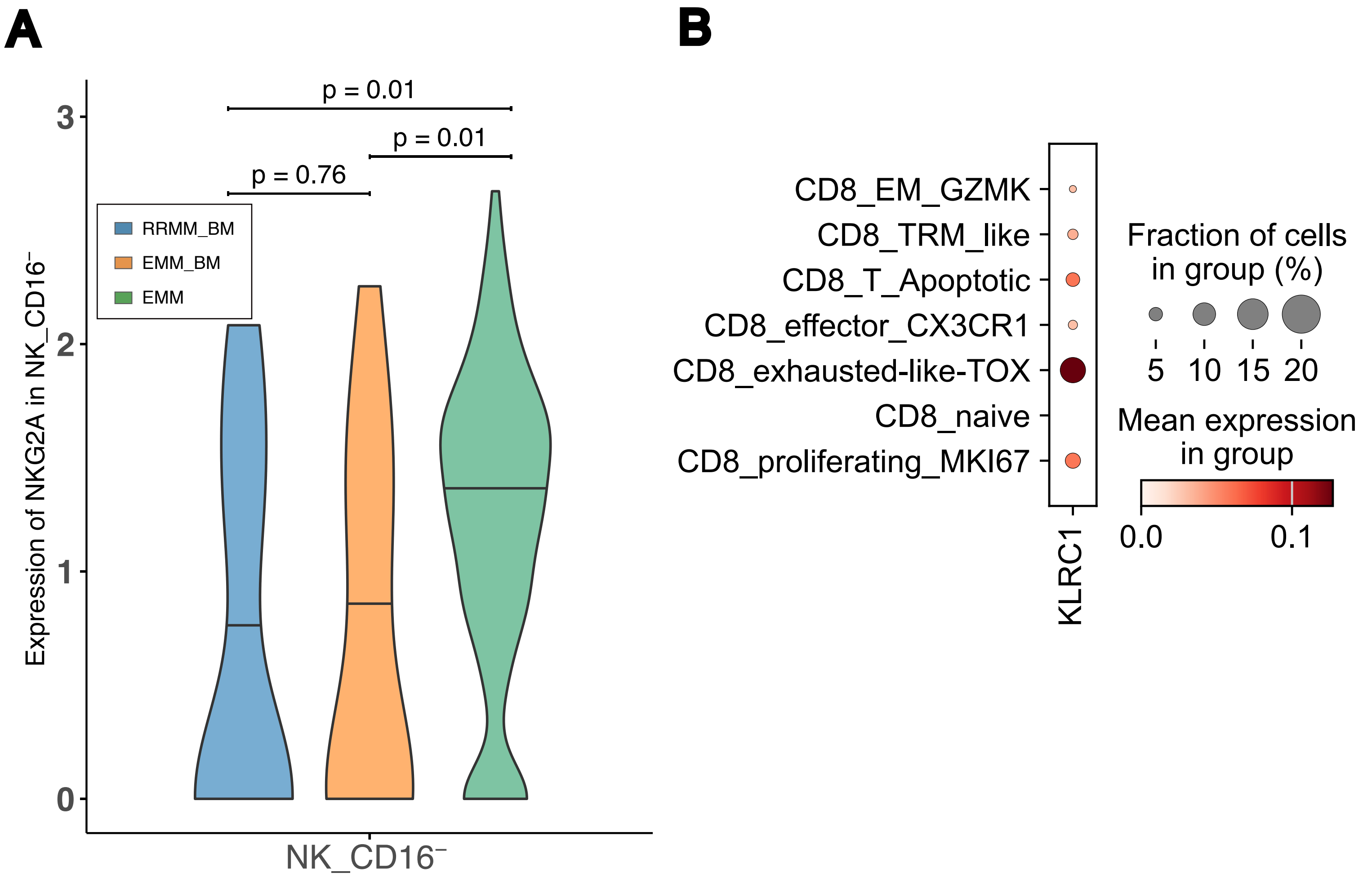

Supplementary Figure 8: Expression of KLRC1(NKG2A): **(A)** Violin plot representing expression of KLRC1 (NKG2A) in CD16- NK cells by scRNAseq **(B)** Dotplot indicating expression of NKG2A across CD8+ T cell subclusters by scRNAseq. Violine plot display the median represented by center line. Statistical comparisons were performed using Wilcoxon rank-sum test with Benjamini–Hochberg correction for multiple testing.
