# Supplementary material for: Composition and Functional State of T and NK Cells in the Extramedullary Myeloma Tumor Microenvironment
Source: Blood Cancer Discov. 2025 Nov 14;7(2):250–65. doi: 10.1158/2643-3230.BCD-25-0170 (PMC13012251; doi:10.1158/2643-3230.BCD-25-0170)
Supplement: Figure S9 — NK cell abundance in spatial data [file bcd-25-0170_figure_s9_suppsf9.pdf]

Supplementary Figure 9

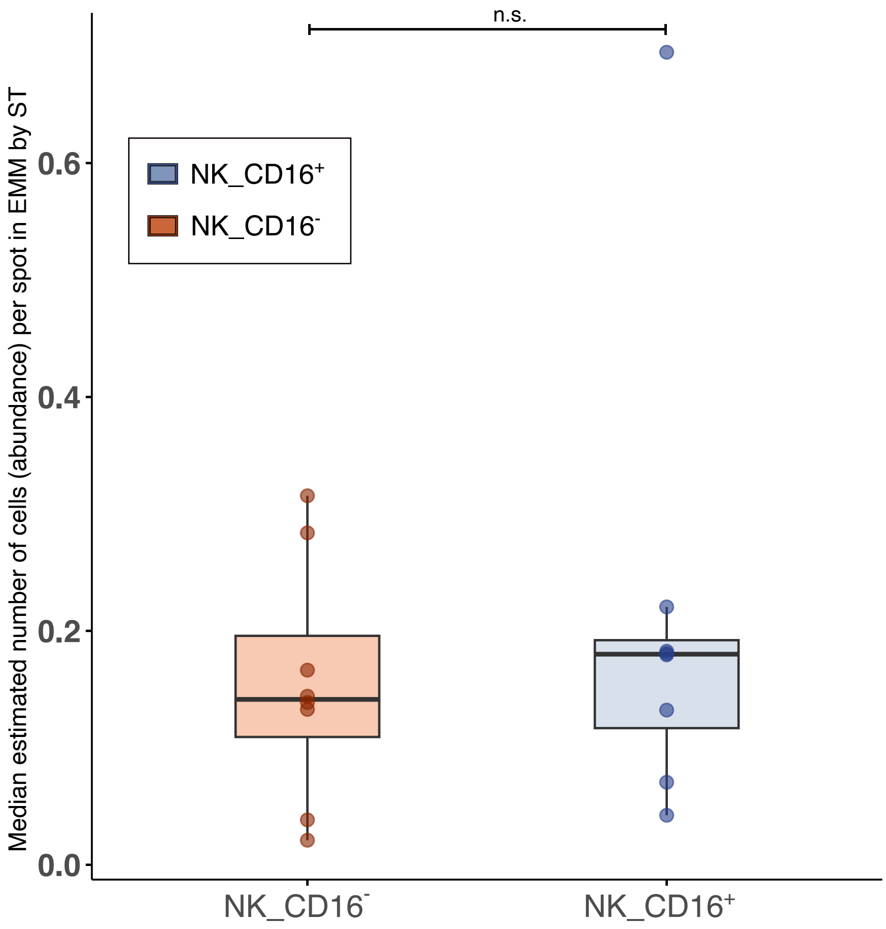

Supplementary Figure 9: NK cell abundance in spatial data: Boxplot comparing median estimated number of cells (abundance) per spot of NK cell subclusters in EMM by spatial transcriptomic data. Boxplots display the median (center line), the 25th and 75th percentiles (box limits), and whiskers extending to the most extreme data points within 1.5× the interquartile range. Statistical comparisons were performed using Wilcoxon rank-sum test with Benjamini–Hochberg correction for multiple testing. n.s. = not significant
