# Supplementary material for: Composition and Functional State of T and NK Cells in the Extramedullary Myeloma Tumor Microenvironment
Source: Blood Cancer Discov. 2025 Nov 14;7(2):250–65. doi: 10.1158/2643-3230.BCD-25-0170 (PMC13012251; doi:10.1158/2643-3230.BCD-25-0170)
Supplement: Figure S10 — Cell type abundances measure by Spatial transcriptomic data [file bcd-25-0170_figure_s10_suppsf10.pdf]

Supplementary Figure 10

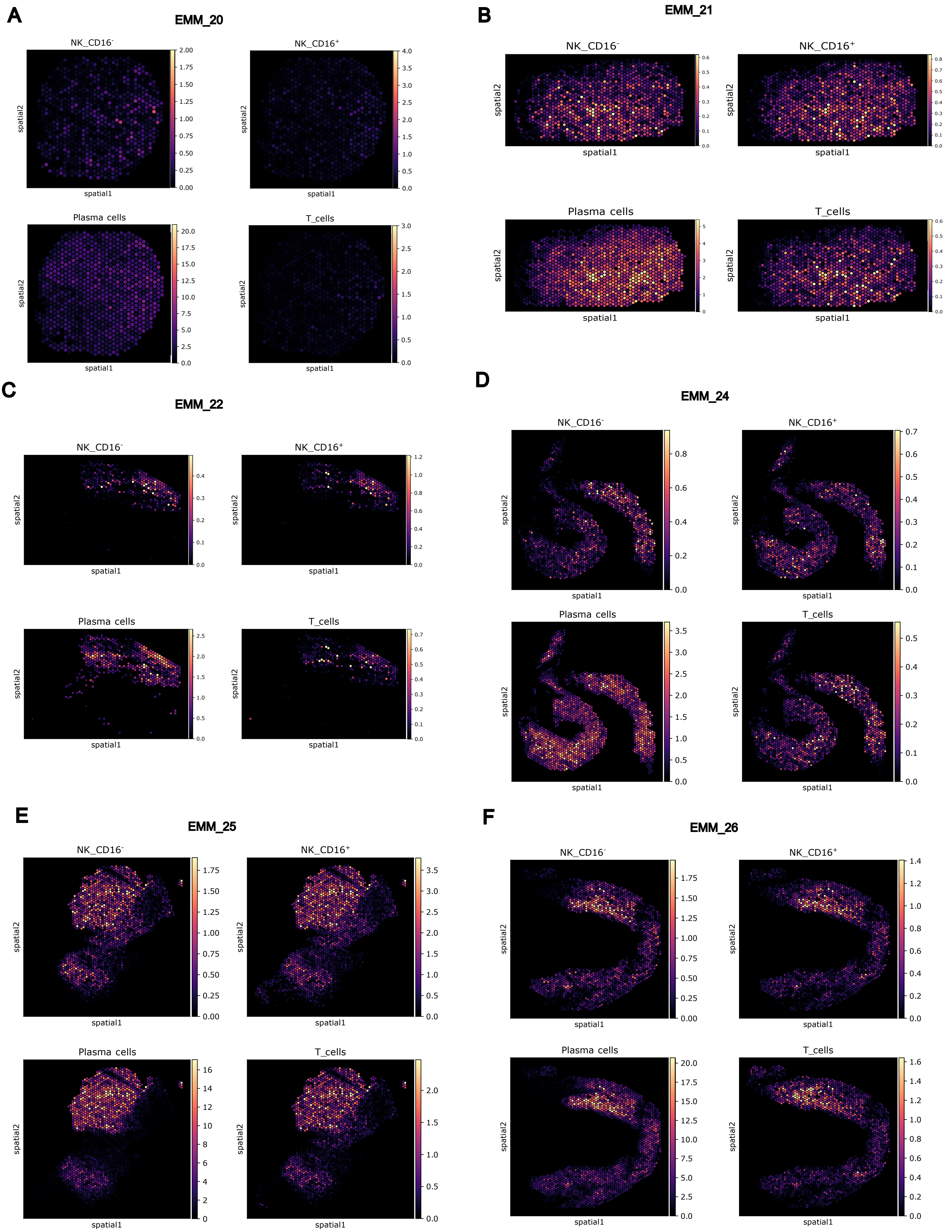

Supplementary Figure 10: Cell type abundances measure by Spatial transcriptomic data: **(A-F)** Estimated cell type abundance of CD16+ and CD16- NK cells, plasma cells and T cells across samples EMM_20, EMM_21, EMM_22, EMM_24, EMM_25 and EMM_26
