# Supplementary material for: Composition and Functional State of T and NK Cells in the Extramedullary Myeloma Tumor Microenvironment
Source: Blood Cancer Discov. 2025 Nov 14;7(2):250–65. doi: 10.1158/2643-3230.BCD-25-0170 (PMC13012251; doi:10.1158/2643-3230.BCD-25-0170)
Supplement: Figure S11 — Myeloid subclusters measure by FCM and scRNAseq [file bcd-25-0170_figure_s11_suppsf11.pdf]

Supplementary Figure 11

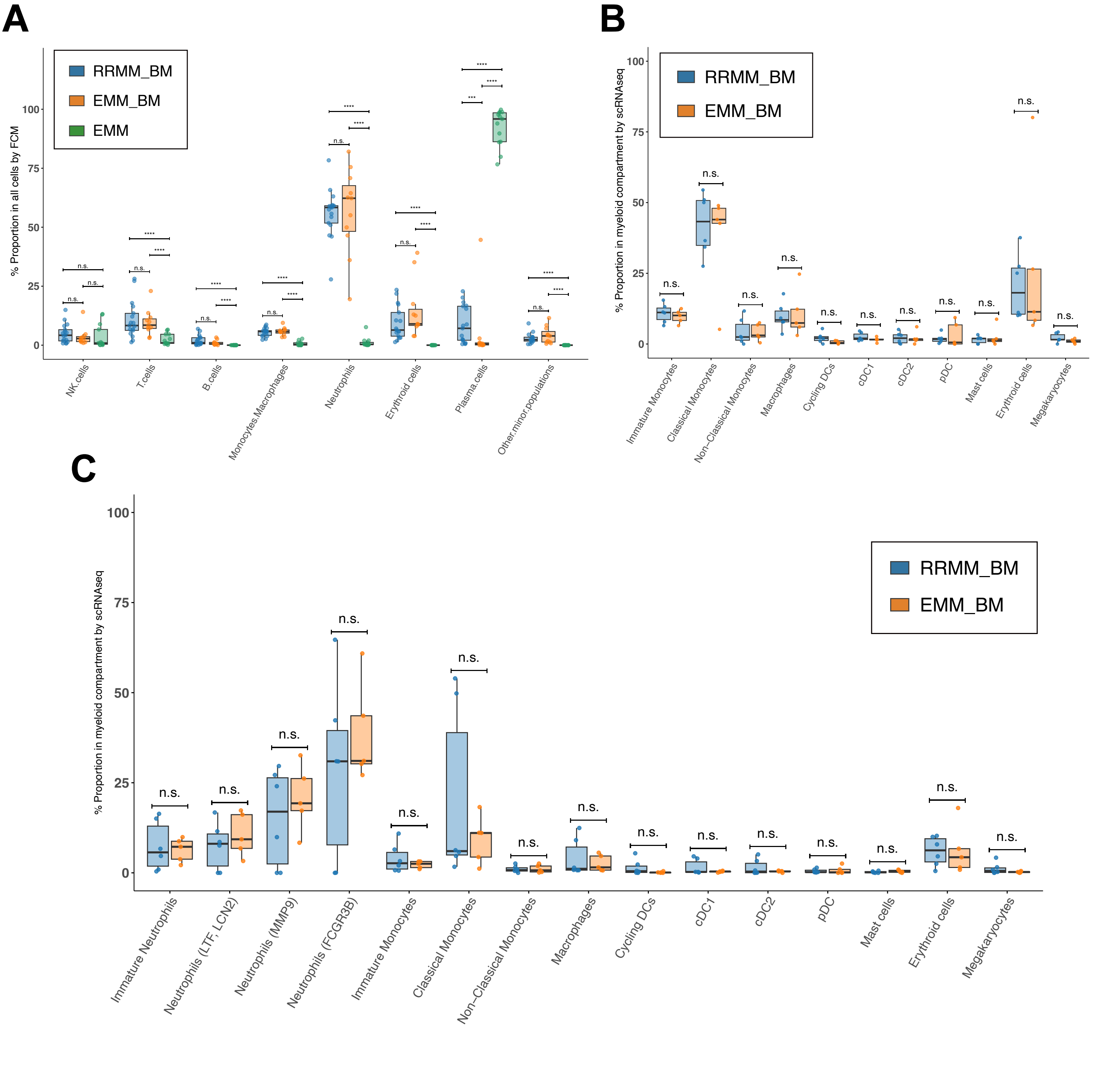

Supplementary Figure 11: Myeloid subclusters measured by FCM and scRNA-seq: Boxplots showing proportion of subclusters identified in **(A)** global comparison of all cell types by FCM and **(B)** myeloid compartment excluding neutrophils by scRNA-seq as a direct comparison between scRNA-seq and FCM results revealed differences in neutrophil levels in few RRMM patients. This discrepancy is likely due to a delay in sample processing for scRNA-seq, as neutrophils are highly sensitive to degradation, contain relatively low levels of RNA, and have high levels of Rnases (https://kb.10xgenomics.com/hc/en-us/articles/360004024032-Can-I-process-neutrophils-or-other-granulocytes-using-10x-Single-Cell-applications). Therefore, we also compared EMM_BM and RRMM_BM after excluding neutrophils, which resulted in no significant differences in myeloid subsets. **(C)** myeloid compartment by scRNA-seq including neutrophils. Samples with < 10 cells in myeloid compartment is excluded from (B) and (C). Boxplots display the median (center line), the 25th and 75th percentiles (box limits), and whiskers extending to the most extreme data points within 1.5× the interquartile range. Statistical comparisons were performed using Wilcoxon rank-sum test with Benjamini–Hochberg correction for multiple testing. n.s = not significant, **p < 0.05; ***p < 0.01; ****p < 0.001
