# Supplementary material for: Composition and Functional State of T and NK Cells in the Extramedullary Myeloma Tumor Microenvironment
Source: Blood Cancer Discov. 2025 Nov 14;7(2):250–65. doi: 10.1158/2643-3230.BCD-25-0170 (PMC13012251; doi:10.1158/2643-3230.BCD-25-0170)
Supplement: Figure S12 — Representative gating strategy of 7-AAD staining [file bcd-25-0170_figure_s12_suppsf12.pdf]

Supplementary Figure 12

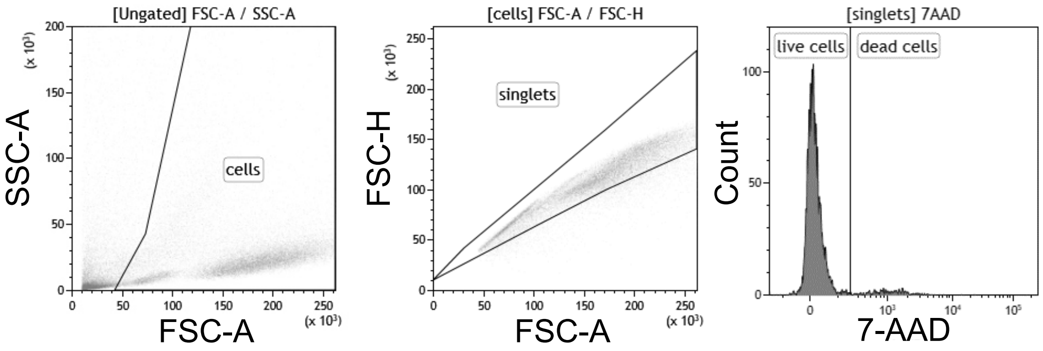

Supplementary Figure 12: Representative gating strategy of 7-AAD staining: To discriminate live cells, samples for scRNA-sequencing were stained with 7-AAD before sorting. Initial gating strategy consisted of excluding debris and doublets using FSC-A/SSC-A and FSC-A/FSC-H dotplots. Subsequently, live cells were gated on histogram as 7-AAD negative.
