# Supplementary material for: Composition and Functional State of T and NK Cells in the Extramedullary Myeloma Tumor Microenvironment
Source: Blood Cancer Discov. 2025 Nov 14;7(2):250–65. doi: 10.1158/2643-3230.BCD-25-0170 (PMC13012251; doi:10.1158/2643-3230.BCD-25-0170)
Supplement: Figure S13 — Batch correction [file bcd-25-0170_figure_s13_suppsf13.pdf]

Supplementary Figure 13

A

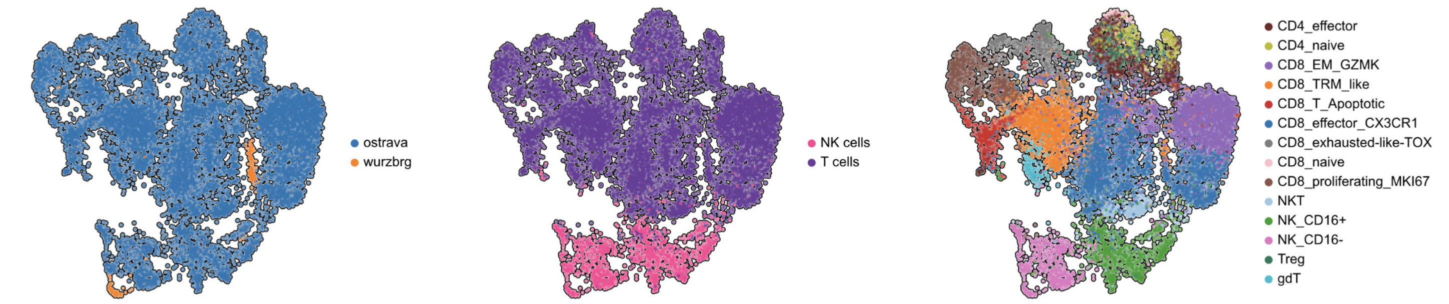

B

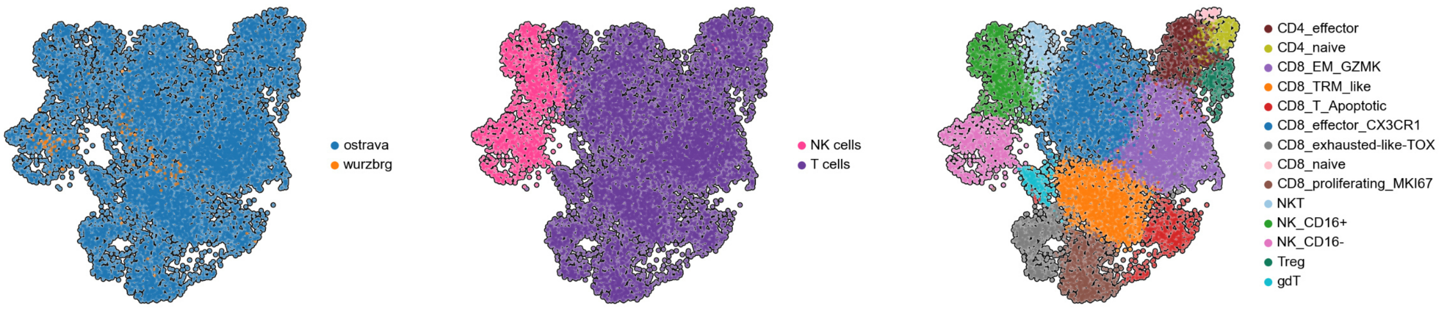

Supplementary Figure 13: Batch correction: UMAP representation of T/NK subcluster from all samples (EMM (N = 7), EMM_BM (N = 5), RRMM_BM (N = 6)) colored by processing center, annotation of T/NK clusters in low and high resolutions **(A)** before batch correction and **(B)** after batch correction
