# Supplementary material for: Composition and Functional State of T and NK Cells in the Extramedullary Myeloma Tumor Microenvironment
Source: Blood Cancer Discov. 2025 Nov 14;7(2):250–65. doi: 10.1158/2643-3230.BCD-25-0170 (PMC13012251; doi:10.1158/2643-3230.BCD-25-0170)
Supplement: Figure S14 — Copy number analysis on EMM (N = 10) and RRMM_BM (N = 5) samples [file bcd-25-0170_figure_s14_suppsf14.pdf]

Supplementary Figure 14

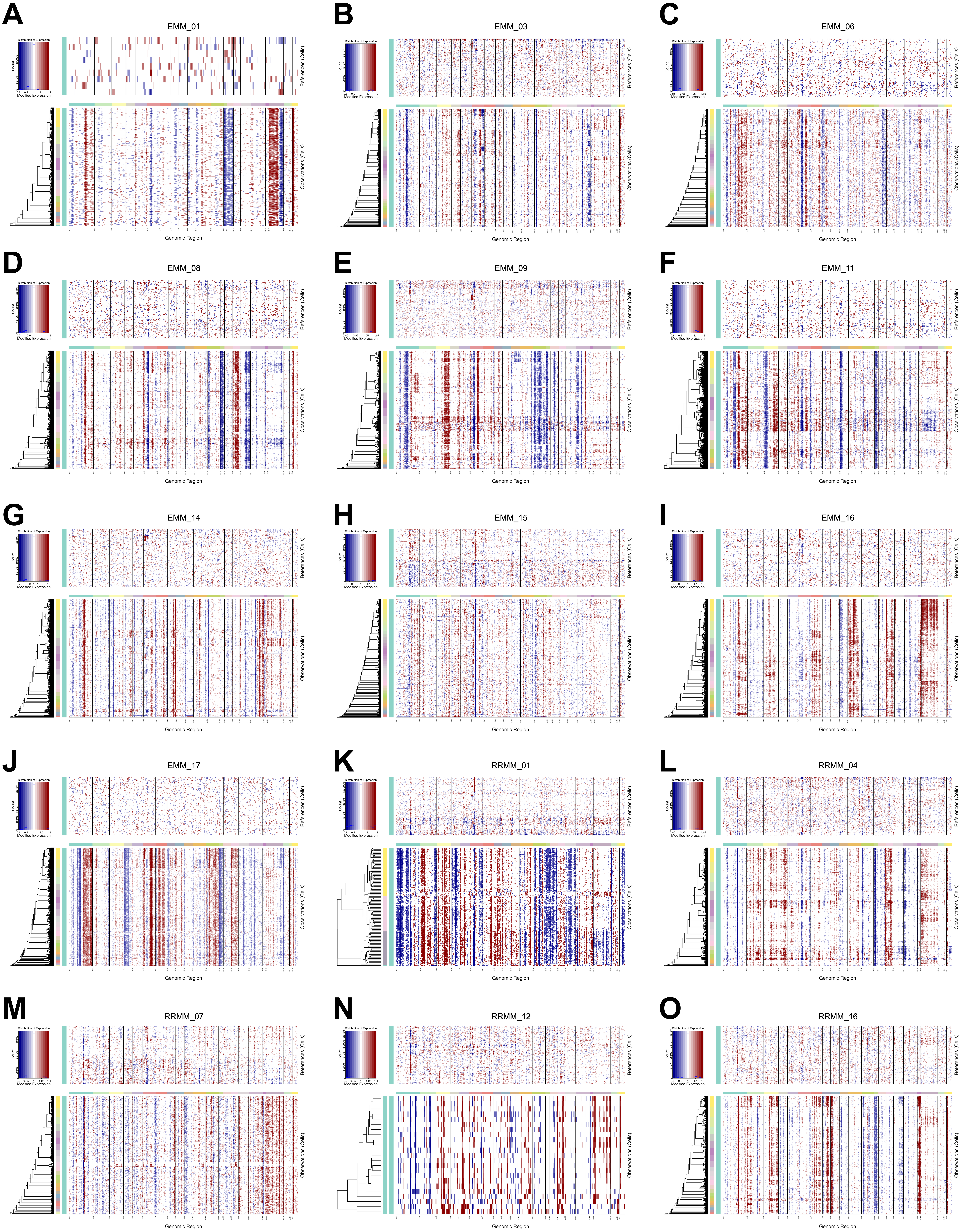

Supplementary Figure 14: Copy number analysis on EMM (N = 10) and RRMM_BM (N = 5) samples: **(A-O)** Heatmap indicating copy number variations in EMM and RRMM_BM samples with atleast 10 plasma cells identified
