# Supplementary material for: Composition and Functional State of T and NK Cells in the Extramedullary Myeloma Tumor Microenvironment
Source: Blood Cancer Discov. 2025 Nov 14;7(2):250–65. doi: 10.1158/2643-3230.BCD-25-0170 (PMC13012251; doi:10.1158/2643-3230.BCD-25-0170)
Supplement: Figure S15 — UMAPs separated by samples [file bcd-25-0170_figure_s15_suppsf15.pdf]

Supplementary Figure 15

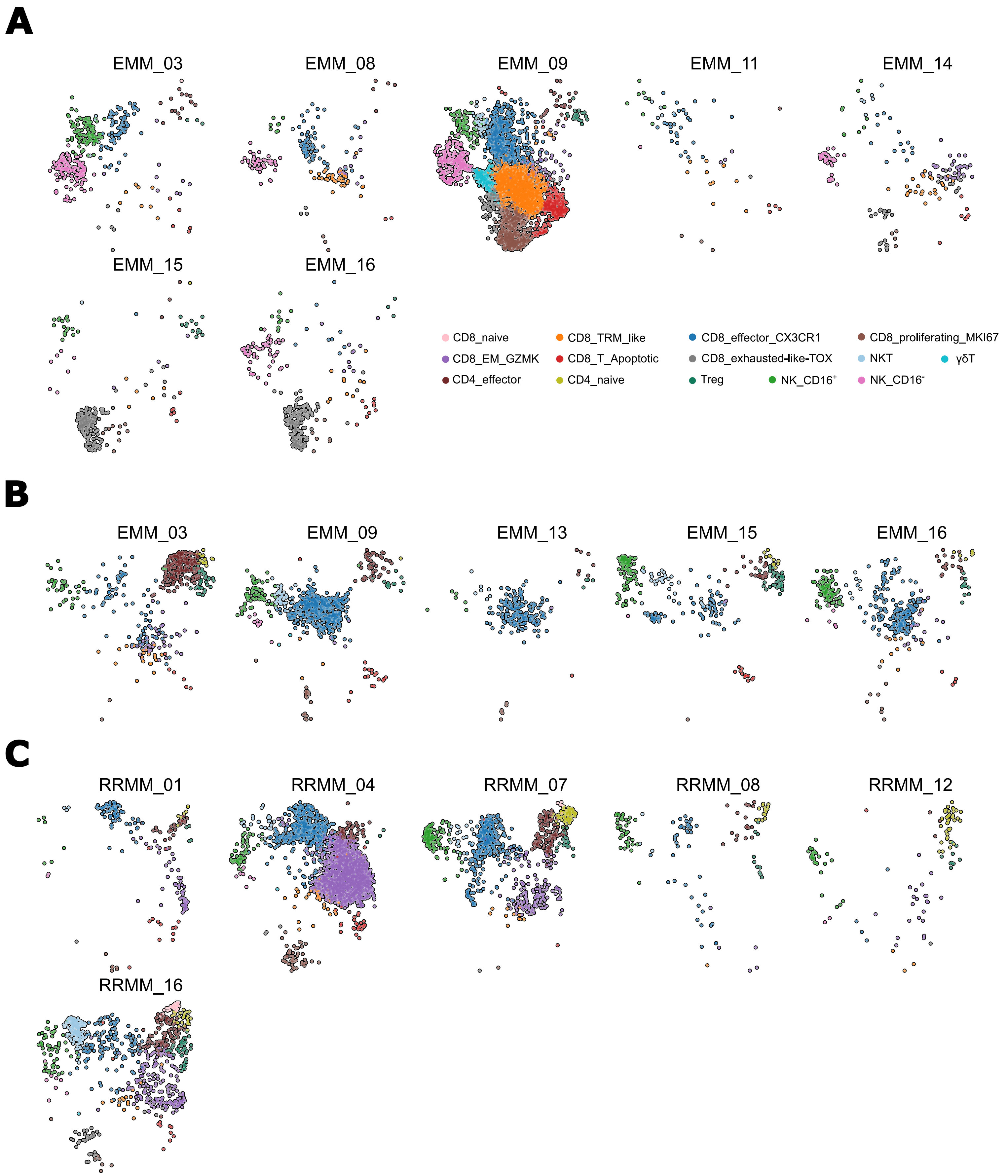

Supplementary Figure 15: UMAPs separated by samples: UMAPS of T/NK subclusters by scRNA-seq separated for each sample in **(A)** EMM **(B)** EMM_BM and **(C)** RRMM_BM cohorts
