# Supplementary material for: Composition and Functional State of T and NK Cells in the Extramedullary Myeloma Tumor Microenvironment
Source: Blood Cancer Discov. 2025 Nov 14;7(2):250–65. doi: 10.1158/2643-3230.BCD-25-0170 (PMC13012251; doi:10.1158/2643-3230.BCD-25-0170)
Supplement: Figure S16 — Representative gating strategy of basic immune subpopulations [file bcd-25-0170_figure_s16_suppsf16.pdf]

# Supplementary Figure 16

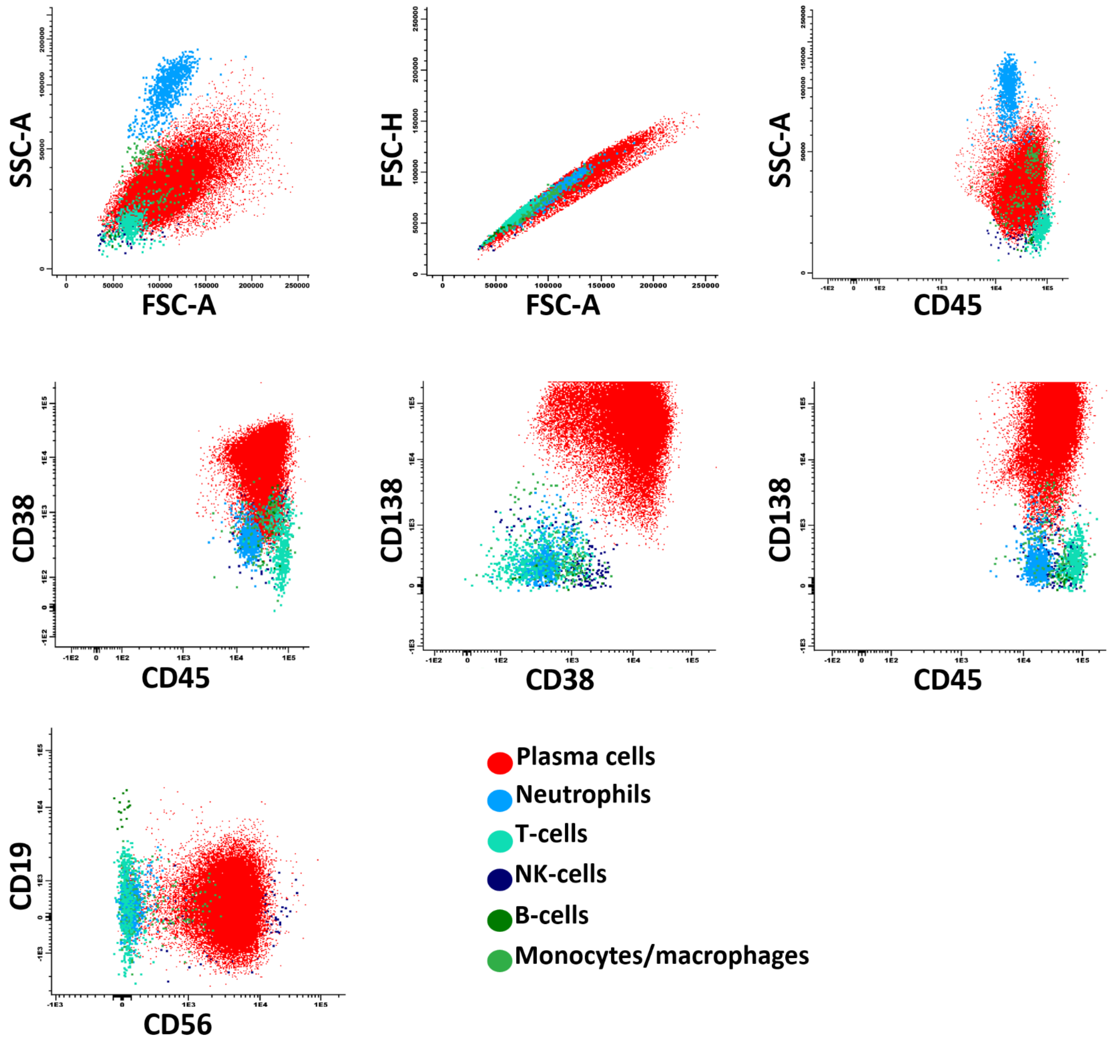


**Supplementary Figure 16:** Representative gating strategy of basic immune subpopulations: For dissection of basic immune subpopulations, EMM samples were stained using stardardized Euroflow 8-color plasma cell disorders (PCD) tube 1. Initial gating strategy consisted of excluding debris and doublets using FSC-A/SSC-A and FSC-A/FSC-H dotplots, followed by gating for $CD138^{+}$ $CD38^{+}$ plasma cells, according to their specific immunophenotype. Subsequently, other cell types were gated according to their immunophenotype or scatter characteristics, neutrophils as $SSC^{high}$, T-cells as $CD19^{-}CD56^{-}$ double negative lymphocytes ($CD45^{+}SSC\text{-}A^{low}$), NK-cells as $CD19^{-}CD56^{+}$ lymphocytes, B-cells as $CD19^{+}CD56^{-}$ lymphocytes and monocytes/macrophages according to their scatter characteristics.
