# Supplementary material for: Composition and Functional State of T and NK Cells in the Extramedullary Myeloma Tumor Microenvironment
Source: Blood Cancer Discov. 2025 Nov 14;7(2):250–65. doi: 10.1158/2643-3230.BCD-25-0170 (PMC13012251; doi:10.1158/2643-3230.BCD-25-0170)
Supplement: Figure S17 — Representative gating strategy of T cells [file bcd-25-0170_figure_s17_suppsf17.pdf]

Supplementary Figure 17

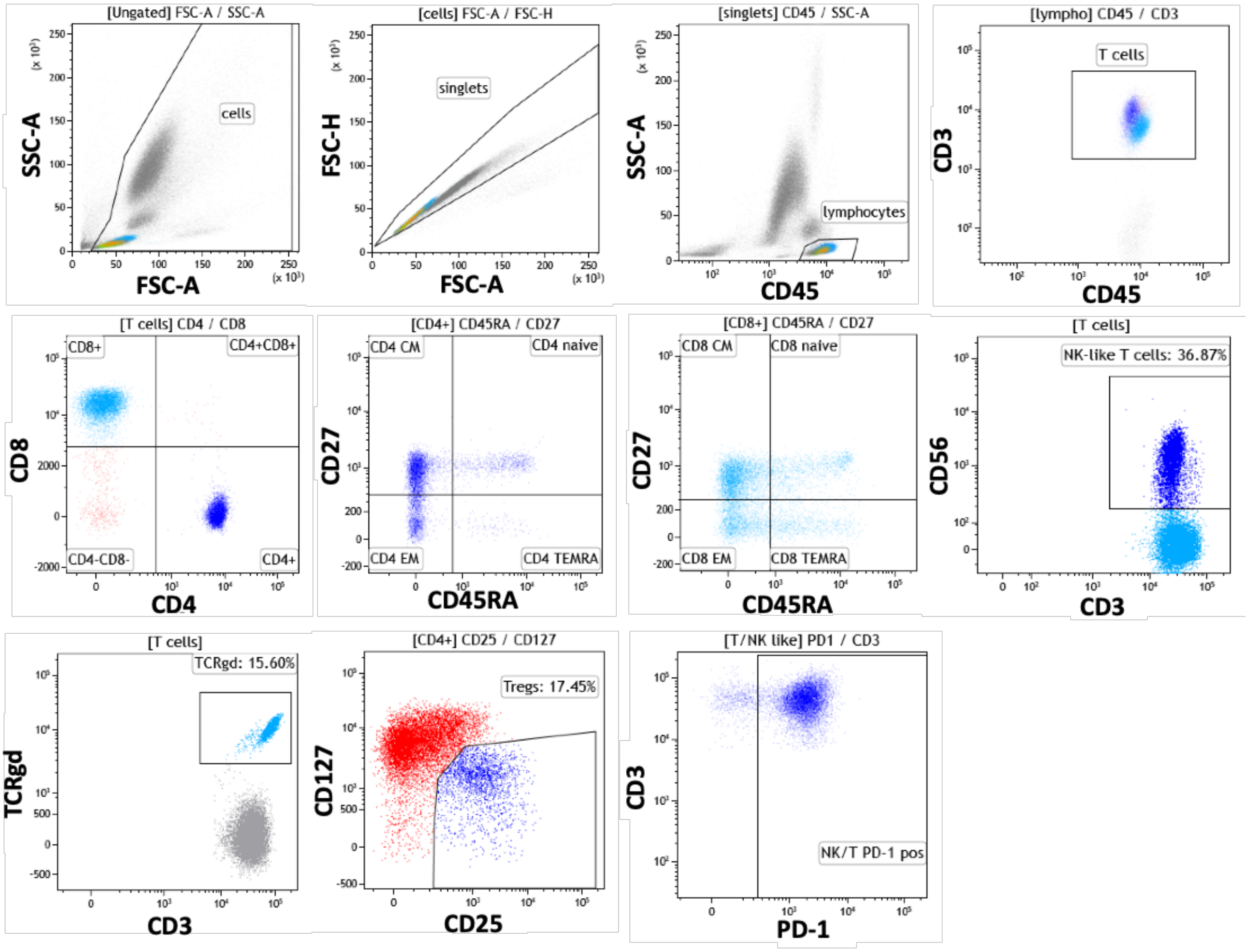

Supplementary Figure 17: Representative gating strategy of T cells: To characterize T-cell subsets in detail, samples were stained using three different tubes: 1. T-CLPD tube 1 to characterize basic subtypes of; 2. LST tube to estimate the level of TCRgd T-cells.; 3. T-reg tube to estimate the level of regulatory T-cells. Initial gating strategy consisted of excluding debris and doublets using FSC-A/SSC-A and FSC-A/FSC-H dotplots, followed by gating for lymphocytes ($CD45^{+}SSC\text{-}A^{low}$). Subsequently, all T-cells ($CD3^{+}$) were gated, out of which $CD4^{+}$, $CD8^{+}$, $CD4^{-}CD8^{-}$ double negative, $CD4^{+}CD8^{+}$ double positive cells were distinguished on CD4/CD8 dotplot. To discriminate between the differentiation stages (naive, central memory (CM), effector memory (EM), terminal effector memory T-cells (TEMRA)) from both $CD4^{+}$ and $CD8^{+}$ T-cells, CD27/CD45RA dotplot was used. NK-like T-cells level was assessed by gating $CD3^{+}CD56^{+}$ double positive cells. The level of regulatory T-cells was evaluated from CD127/CD25 dotplot. Additionally, the level of T-cells positive for PD-1 was evaluated.
