# Supplementary material for: Composition and Functional State of T and NK Cells in the Extramedullary Myeloma Tumor Microenvironment
Source: Blood Cancer Discov. 2025 Nov 14;7(2):250–65. doi: 10.1158/2643-3230.BCD-25-0170 (PMC13012251; doi:10.1158/2643-3230.BCD-25-0170)
Supplement: Figure S18 — Representative gating strategy of NK cells [file bcd-25-0170_figure_s18_suppsf18.pdf]

Supplementary Figure 18

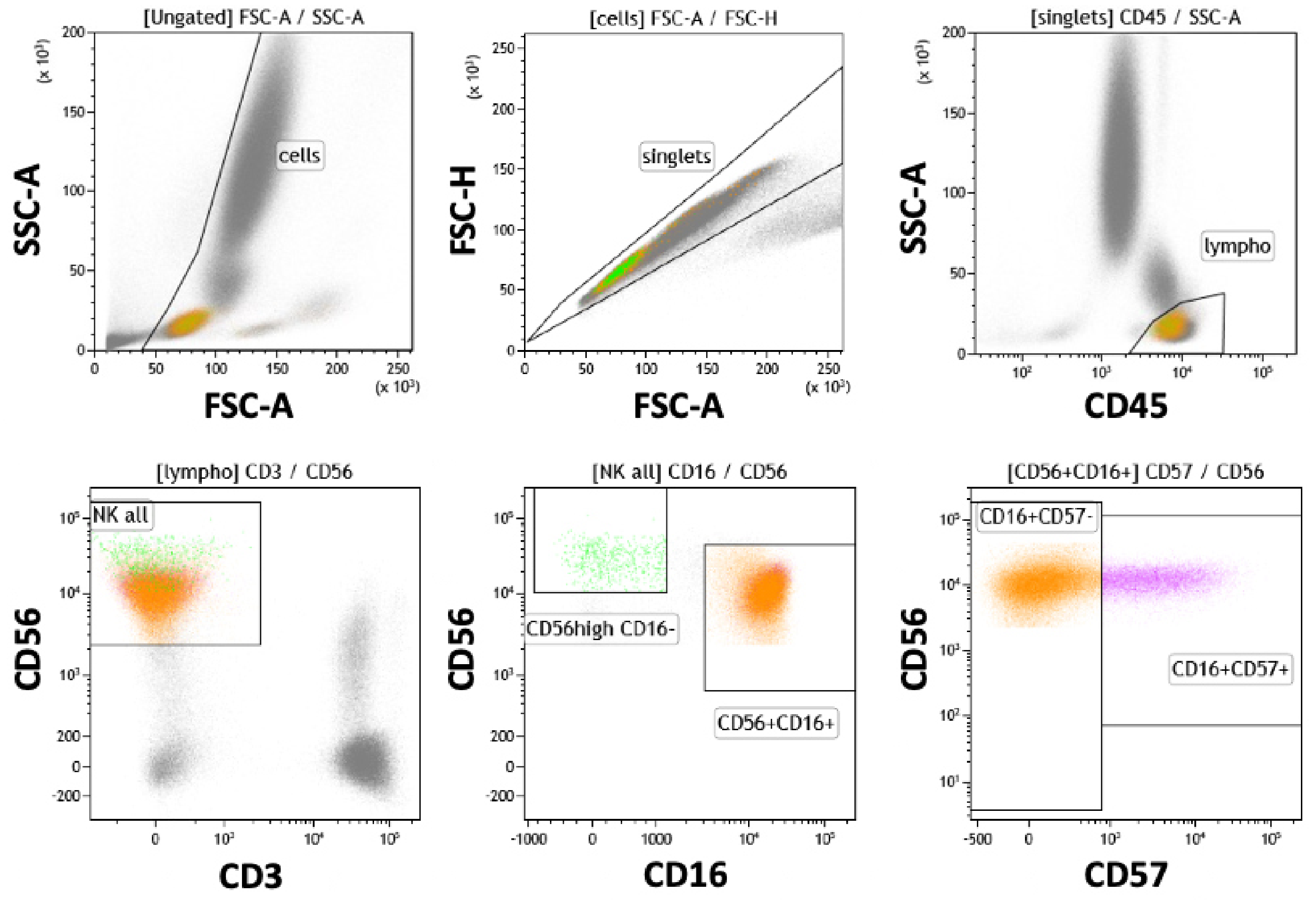

Supplementary Figure 18: Representative gating strategy of NK cells: To characterize NK-cell subsets in detail samples were stained with two 8-color tubes, containing several markers of activation/inhibition. Initial gating strategy consisted of excluding debris and doublets using FSC-A/SSC-A and FSC-A/FSC-H dotplots, followed by gating for lymphocytes ($CD45^{+}SSC\text{-}A^{low}$). Subsequently, all NK-cells were gated ($CD56^{+}CD3^{-}$), out of which $CD56^{high}CD16^{-}$ and $CD56^{+}CD16^{+}$ NK-cells were distinguished using CD56/CD16 dotplot. Next, $CD56^{+}CD16^{+}CD57^{-}$ and $CD56^{+}CD16^{+}CD57^{+}$ NK-cells were gated using CD56/CD57 dotplot. Finally, the level of NK-cells positive for NKG2A checkpoint molecule was evaluated.
